# Oropouche virus causes acute hepatitis in mice controlled by type I interferons

**DOI:** 10.1128/jvi.00611-26

**Published:** 2026-06-30

**Authors:** Cade E. Sterling, Rachael E. Rush, Jackson J. McGaughey, Brooke A. Snow, Alexandra J. Benton, W. Paul Duprex, Gaya K. Amarasinghe, Satdarshan P. Monga, Amy L. Hartman

**Affiliations:** 1 Center for Vaccine Research, University of Pittsburgh School of Medicine 12317, Pittsburgh, Pennsylvania, USA; 2 Department of Infectious Disease and Microbiology, University of Pittsburgh School of Public Health 51303, Pittsburgh, Pennsylvania, USA; 3 Department of Pathology and Immunology, Washington University School of Medicine in St. Louis 12275, St. Louis, Missouri, USA; 4 Department of Pharmacology, Department of Medicine, Pittsburgh Liver Research Center, Organ Pathobiology and Therapeutic Institute, University of Pittsburgh School of Medicine 12317, Pittsburgh, Pennsylvania, USA; University of Minnesota Twin Cities, Minneapolis, Minnesota, USA

**Keywords:** viral hepatitis, mouse model, peribunyavirus, bunyavirus, oropouche virus

## Abstract

Oropouche virus (OROV), a member of the *Peribunyaviridae* family endemic to South America, is a current public health threat. The recent OROV outbreak driven by a novel reassortant strain has caused a dramatic increase in cases in 2024 (13,785 in Brazil, versus only 261 from 2015 to 2022) with sustained levels of transmission in 2025. Previously underreported outcomes have been recognized, including miscarriage, microcephaly, encephalitis, and death. OROV lethality in humans has been attributed to severe coagulopathy with liver involvement, and epidemiological data suggest that acute hepatitis occurs in mild cases of Oropouche fever, highlighting the underrecognized role of the liver in OROV pathogenesis. We present two discrete mouse models of OROV hepatic disease—a lethal model that is similar to the severe coagulative liver necrosis seen in fatal human cases and a model of self-resolving acute hepatitis, which is similar to mild human disease. In both models, OROV causes focal hepatic necrosis in mice, which progresses to massive necrosis and death when the Type I interferon receptor is antagonized. Additionally, we found that a contemporary OROV isolate is less pathogenic in mice than a historic prototypical strain. These studies enhance our understanding of OROV pathogenesis and provide additional models for potential therapeutic development and evaluation.

**IMPORTANCE:**

The disease burden of Oropouche fever has been underrecognized and underreported, as highlighted by the increased testing seen in an ongoing outbreak in South America. Specifically, the role of the liver in Oropouche virus pathogenesis has been understudied. Given the recent increase in cases and severe disease manifestations, there is a present need to understand Oropouche virus pathogenesis and provide models to test potential therapeutics. The mouse models of Oropouche-induced hepatitis presented here provide a means to understand how Oropouche virus causes liver damage in both a lethal and sublethal context. These models will be useful for the preclinical evaluation of vaccines and therapeutic treatments. Additionally, we compare the pathogenicity of a historical Oropouche virus isolate to a contemporary human isolate in a lethal mouse model.

## INTRODUCTION

Oropouche virus (OROV; class *Bunyaviricetes*, family *Peribunyaviridae*, Simbu serogroup) causes sporadic human outbreaks, primarily in the Amazon Basin, where spillover occurs from sloths, non-human primates, and birds via *Culicoides* midges or, to a lesser extent, *Culex* mosquitoes (1). In humans, Oropouche fever most often presents as a self-limiting febrile illness characterized by headaches, myalgia, joint pain, and gastrointestinal distress, though occasional cases of severe neurologic disease have been reported (2–4).

The largest recorded OROV outbreak is currently ongoing with over 16,000 confirmed cases in the Americas in 2024 alone and a resurgence of cases in 2025 at similar levels (5–8). Genomic analyses of OROV cases from August 2022 to February 2024 discovered that this outbreak coincided with the emergence of a novel reassortant lineage of OROV (9). Brazil endured the largest disease burden with 831 reported cases in 2023 and 13,785 cases in 2024 (compared to only 216 total cases between 2015 and 2022) (7, 10, 11). Imported travel-associated OROV cases were found in non-endemic regions including the United States, Italy, Spain, and Germany (12–14). Moreover, there has been OROV emergence and ongoing transmission in Cuba, highlighting the ability of OROV to establish transmission in new regions (15, 16). Notably, competent vectors are found in the United States, Canada, and Europe (13, 17–20).

Beyond increased transmission and reported cases, the 2023–2025 outbreak resulted in recognition of previously unreported forms of clinical disease including adverse pregnancy outcomes (i.e., *in utero* transmission, fetal loss, and microcephaly), severe neurologic outcomes in adults (i.e., Guillain-Barré syndrome), hemorrhagic fever, and death (5, 12, 15, 21–25). Prior to this outbreak, there were no known cases of lethal Oropouche fever. It is unclear whether the reassortant strain currently circulating is uniquely more virulent or if the increase in cases and testing has revealed previously undocumented severe outcomes. Taken together, the threat OROV poses to the Americas is apparent, reflected by the fact that the Pan American Health Organization (a World Health Organization regional office) issued a Public Health Risk Assessment rating the regional risk as high (6). Consequently, there is a present need to understand the severe outcomes of Oropouche fever and test potential therapeutics. To this end, the development of relevant animal models is paramount.

The cause of death in the first two lethal human cases of Oropouche fever, which occurred in otherwise healthy individuals, was attributed to severe coagulopathy with liver involvement (5, 22). The third lethal case resulted from severe coagulopathy, respiratory distress, and renal failure; liver histology showed signs of necrotic foci, steatosis, and severe hepatic damage (26). More recent case studies have also documented Oropouche-associated hepatitis in otherwise healthy patients with elevated hepatic transaminases (27, 28). While it has been previously reported that mild cases of Oropouche fever can coincide with mild liver injury, the role of the liver in OROV pathogenesis remains largely unexplored (3).

Here, we report two mouse models of OROV hepatic disease—a sublethal and lethal model. In the sublethal model, immunocompetent mice develop an acute hepatitis characterized by elevated liver enzymes, necrotic foci, and robust OROV replication in the liver. In the lethal model, OROV susceptibility is induced by administration of an anti-Ifnar1 antibody, which results in mice experiencing lethal hepatic disease characterized by sinusoidal congestion and coagulative necrosis in the liver, much like the lethal human cases of Oropouche fever. The development and characterization of these models will be useful for testing vaccines, therapeutics, and other medical countermeasures against OROV.

## RESULTS

### OROV induces non-lethal transient acute hepatitis in immunocompetent mice

Immunocompetent adult laboratory mice do not generally show visible signs of disease following OROV infection from peripheral inoculation routes (intradermal, subcutaneous, or intraperitoneal). In the interest of identifying a lethal infection model that could be used for countermeasure testing, we infected immunocompetent C57BL/6 mice with recombinant OROV BeAn19991 by several routes of infection, including footpad, intraperitoneal, and intranasal inoculation. Indeed, in our hands, infection of mice with 10^6^ PFU of OROV BeAn19991 by footpad injection resulted in sublethal infection with no clinical signs of disease, including a lack of substantial weight loss (Fig. S1). Despite intranasal infection not being a physiologically relevant infection route, inoculation of mice in this manner resulted in lethality at a dose of 10^4^ PFU (Fig. S1). These results demonstrate that this strain of OROV can replicate in mice, and the lack of lethality after footpad infection is due to control of viral infection in the periphery rather than a lack of permissivity to infection.

**Fig 1:**
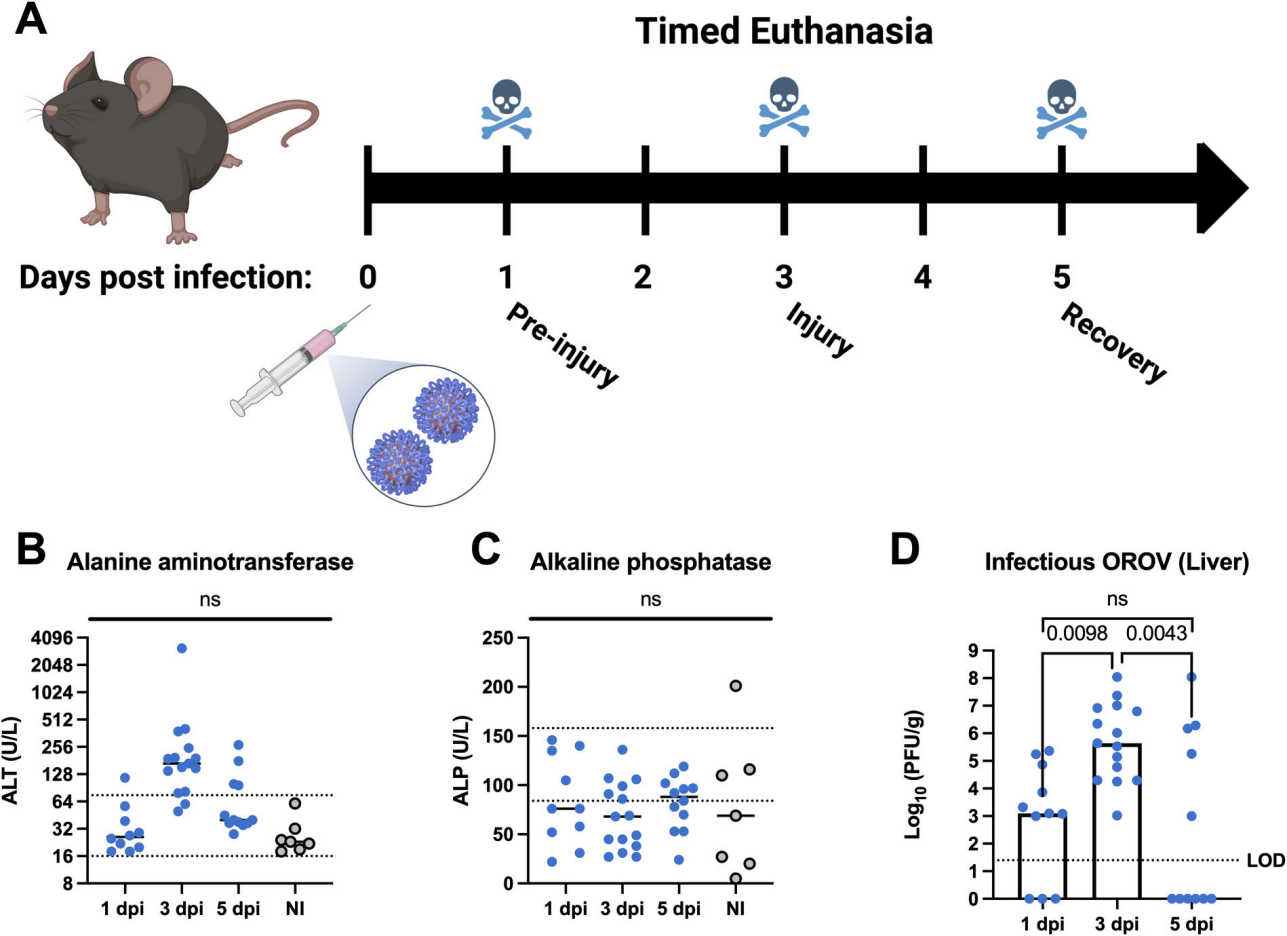
OROV induces transient acute liver injury in immunocompetent mice. (**A**) Schematic of OROV acute hepatitis model. Mice were infected with 10^6^ PFU OROV via footpad injection, and a subset was euthanized at 1 (*n* = 11), 3 (*n* = 15), and 5 (*n* = 11) dpi. (**B**) Blood alanine aminotransferase (ALT) and (**C**) alkaline phosphatase (ALP) levels at indicated time points post-infection or uninfected controls (*n* = 7). (**D**) Infectious OROV liver titers by plaque assay at indicated time point post-infection. Each data point represents one mouse across three experiments; bars represent median value. Dotted lines indicate either normal (95% confidence interval) range or limit of detection (LOD). Statistical significance was determined by ordinary one-way ANOVA and Tukey’s multiple comparisons test. *P*-value shown or ns (not significant) if *P* > 0.05. Uninfected controls labeled “NI” (not infected).

We conducted all further studies herein using the footpad infection route with 10^6^ PFU of the prototypical OROV strain BeAn19991, including a planned euthanasia experiment to determine whether OROV replicates systemically despite lack of outward signs of disease. We euthanized a subset of animals at 1 (*n* = 11), 3 (*n* = 15), and 5 (*n* = 11) days post-infection (dpi; Fig. 1A). Upon necropsy, tissues were harvested for measurement of viral load and histology. At 3 dpi, OROV-infected mice had transiently elevated levels of alanine transaminase (ALT), but not alkaline phosphatase (ALP) or other parameters (Fig. 1B and C and Fig. S2); these blood chemistry results are indicative of mild hepatocellular damage. Median blood chemistry parameters were within the normal range (95% CI for C57BL/6J mice) at 1 and 5 dpi (29). While the elevation at 3 dpi is not statistically significant, the median elevation seen is clinically relevant (sevenfold increase over uninfected controls and over double the upper limit of normal). Infectious OROV was detectable in the liver as early as 1 dpi, with peak titers at 3 dpi (10^3^–10^8^ PFU/g); by 5 dpi, most mice returned to undetectable levels of infectious virus (Fig. 1D). We found no infectious virus in the spleen, kidney, lung, or brain at any time point, and low to moderate levels of viral RNA were detected in these tissues (Fig. S3). Taken together, these data suggest that inapparent OROV infection is hepatotropic in C57BL/6 mice and causes a self-resolving acute hepatitis.

**Fig 2:**
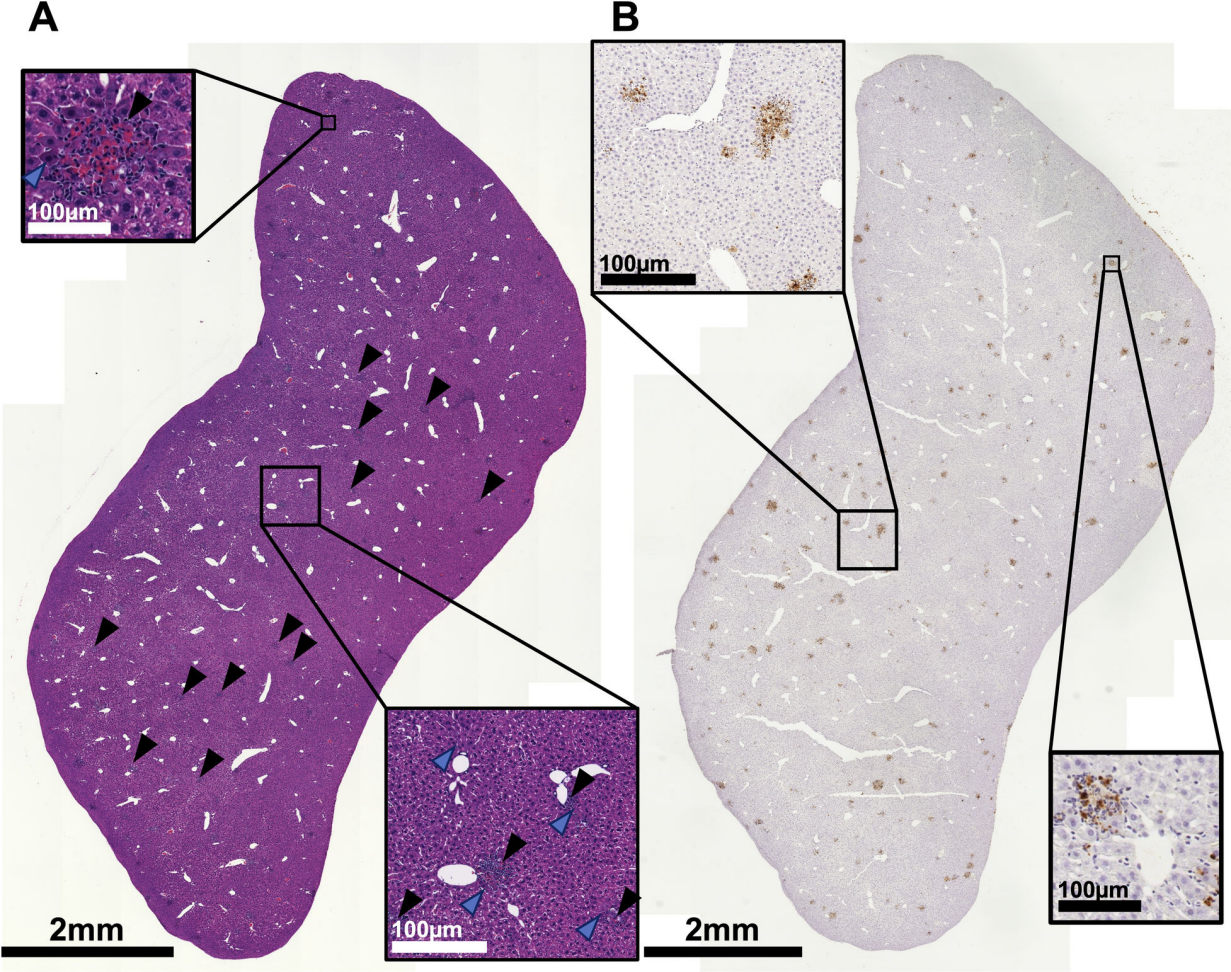
Histopathology reveals OROV-induced necroinflammatory foci across the entire lobule. (**A**) H&E and (**B**) TUNEL staining of a representative FFPE liver left lateral lobe from a mouse euthanized 3 days post-infection (10^6^ PFU inoculation dose; total *n* = 15, three independent experiments). Composite images, a representative 20× region of interest (ROI; 594 × 594 µm), and a magnified inset are shown. Black arrows mark necroinflammatory foci; blue arrows mark regions of swollen hepatocytes. Not all features are marked.

**Fig 3:**
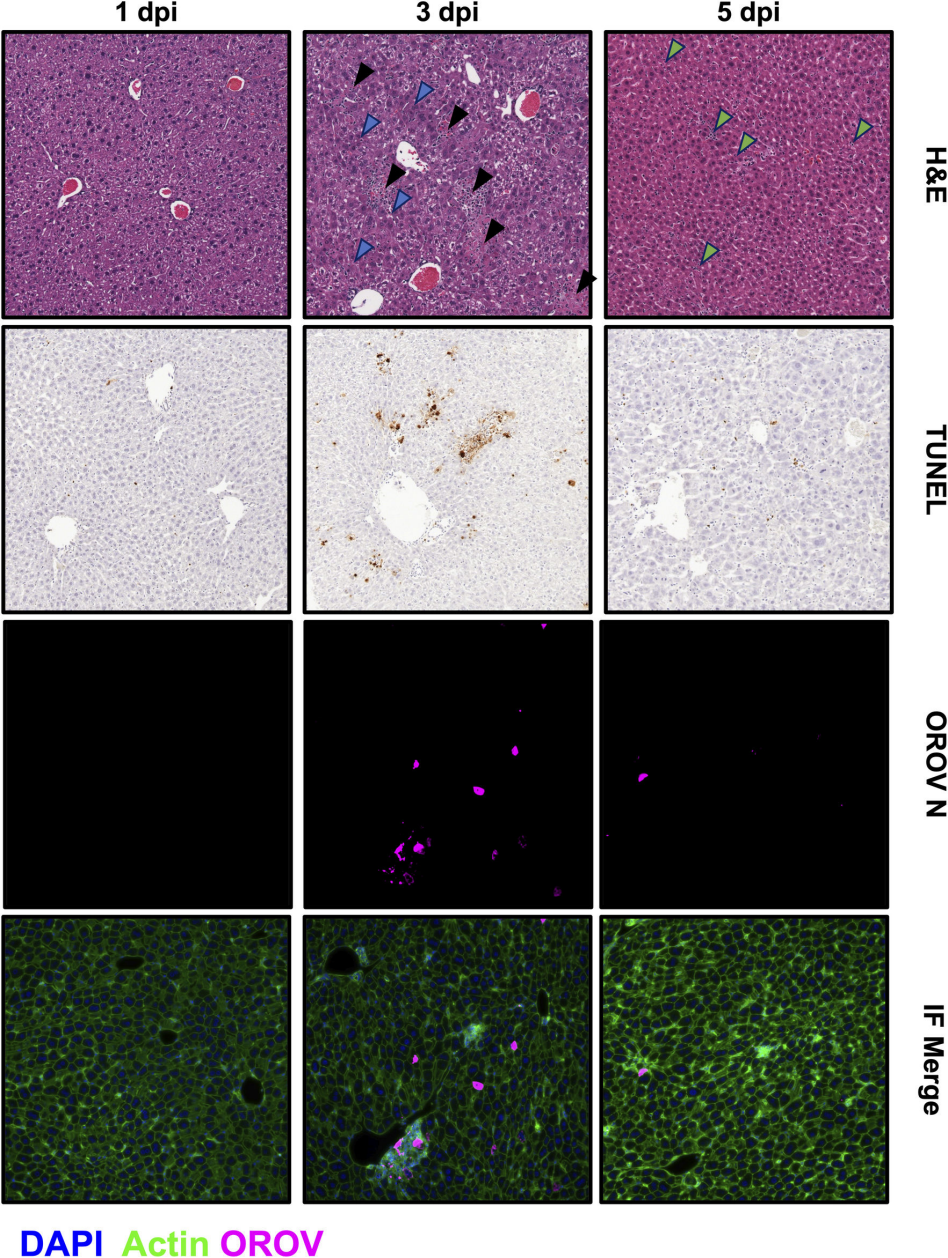
OROV-induced hepatic injury is transient. Representative 20× regions of interest (ROIs) of H&E (594 × 594 µm), TUNEL (594 × 594 µm), and OROV N (670 × 670 µm) staining at 1 (*n* = 11), 3 (*n* = 15), and 5 (*n* = 11) dpi following infection with 10^6^ PFU OROV via footpad injection. H&E and TUNEL staining from FFPE left lateral lobes; OROV N staining from cryo-embedded and sectioned right medial lobes. Labels indicate time point and stain. In H&E images, black arrows mark necroinflammatory foci; blue arrows mark regions of swollen hepatocytes; green arrows mark non-focal immune infiltrates. Not all features are marked.

Given that we found considerable viral titers in the liver along with elevated liver enzymes at 3 dpi, we examined liver tissue for histological damage. Sections of the left lateral lobe were stained by hematoxylin and eosin (H&E), which revealed discrete inflammatory foci (black arrows) throughout the lobule (Fig. 2A). These foci were characterized by a breakdown in cellular architecture, the accumulation of eosin staining (either blood or cytoplasmic accumulation), and a relatively high number of immune infiltrates (the small nuclei seen in the foci; Fig. 2A). The foci were often surrounded by abnormally swollen hepatocytes (blue arrows), which is a non-specific marker of injury (Fig. 2A). To determine if these foci were also areas of cell death, we performed TUNEL staining and found that inflammatory foci were highly TUNEL-positive (Fig. 2B). The degree of ALT elevation at 3 dpi varied between animals (Fig. 1B), and the total area of TUNEL staining across the lobule generally reflected the relative ALT levels (i.e., animals with higher blood ALT levels had more extensive hepatic necrosis; Fig. S4). By immunofluorescent staining, these foci were also positive for OROV N antigen (Fig. 3), suggesting they are sites of viral infection. It is unclear whether the accumulation of OROV antigen is the result of direct infection of hepatocytes that formerly occupied that space or if the infiltrating cells are infected themselves. Based on histopathologic examination, necroinflammatory foci appear to be stochastically distributed across the liver (Fig. 2); however, there may be some preference for midzone (zone 2) and pericentral zone (zone 3) infection as judged by the relationship of these foci to portal triads or central veins. Further investigation into OROV zonal preference is warranted, as the observations here are similar to a human case that reported focal infection with a preference for zones 2 and 3 in the patient’s liver (26).

We also examined whole liver lobules for histological damage at 1 and 5 dpi. Generally, the liver at 1 dpi was indicative of pre-injury, whereas recovery was ongoing at 5 dpi (Fig. 3 and Fig. S5). While low levels of infectious virus were detected in the liver at 1 dpi (Fig. 1D), there is a lack of histopathological damage and OROV antigen at this time (Fig. 3, left column). By 5 dpi, there was less infectious virus (Fig. 1D), OROV antigen, and the necroinflammatory foci had resolved; however, there were still observable histopathological changes (Fig. 3, right column). Specifically, the recovering livers (5 dpi) showed an overall increase in infiltrating immune cells dispersed throughout the liver (green arrows in H&E panel), suggestive of ongoing repair. Notably, there was a lack of focal damage or accumulation of immune cells in foci at 5 dpi (Fig. 3). While there was some increased TUNEL staining at 5 dpi compared to 1 dpi and uninfected controls, it is remarkably less than 3 dpi, further suggesting ongoing repair (Fig. 3 and Fig. S5 and S6 and Fig. S7).

### Anti-IFNAR1 antibody treatment induces OROV susceptibility in wild-type mice

**Fig 4:**
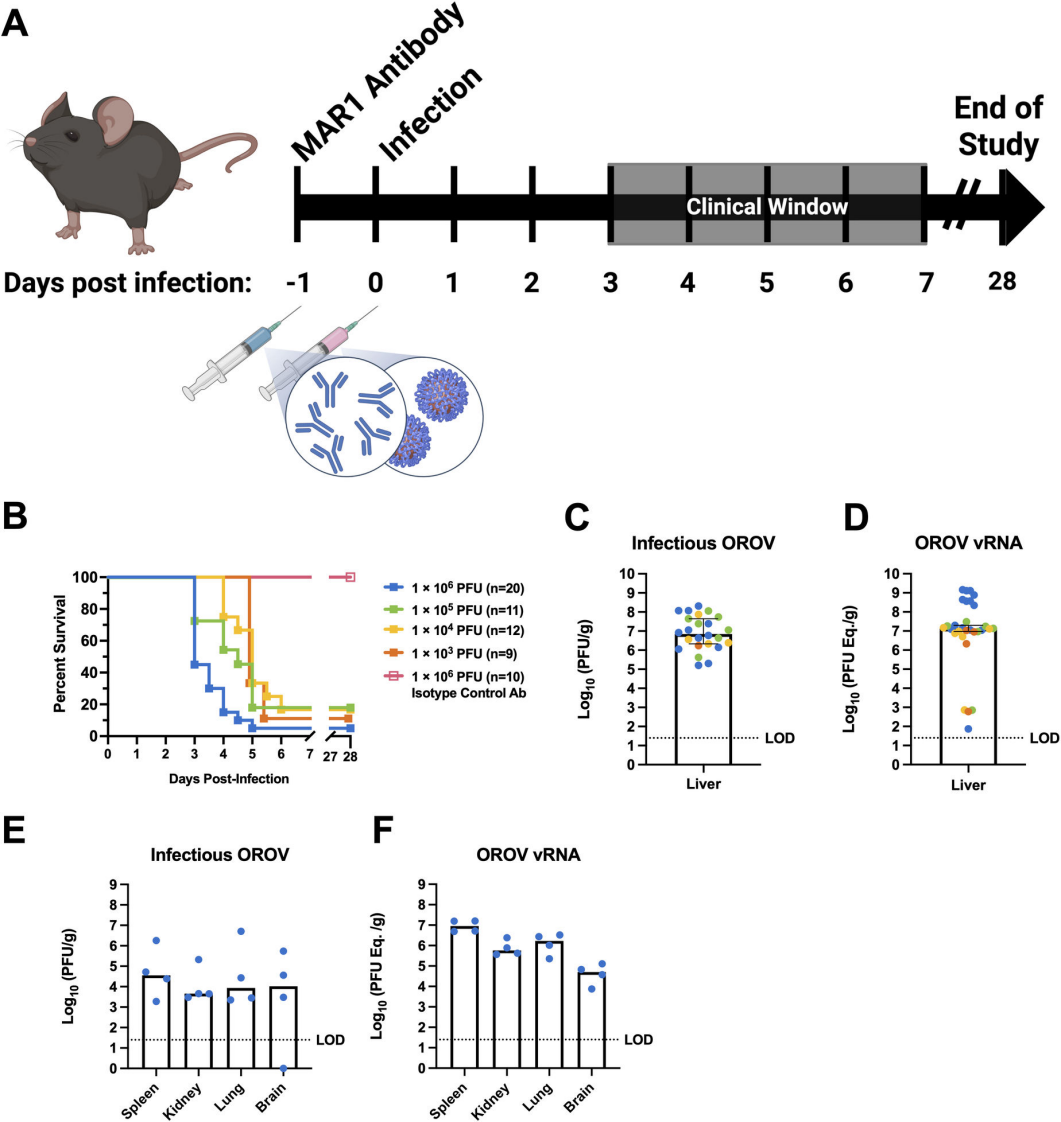
MAR1 antibody treatment induces OROV susceptibility in mice. (**A**) Schematic of lethal OROV MAR1 model. Mice were treated with 500 µg MAR1 Ab 1 day prior to OROV infection via intradermal footpad injection. (**B**) Combined survival curve from five independent experiments. (**C**) Infectious OROV titers in liver homogenate from tissues collected at endpoint. Figure excludes samples unquantifiable due to degradation. Symbols are color-coded based on infection dose. (**D**) Viral RNA in liver homogenate from tissues collected at endpoint was quantified by RT-qPCR. (**E**) OROV titers by plaque assay or (**F**) RT-qPCR in spleen, kidney, lung, and brain tissues from a subset of mice (*n* = 4) at endpoint. Bars represent median values. Error bars represent 95% confidence interval. Dotted lines indicate limit of detection (LOD).

Using an anti-mouse IFNAR1 antibody to transiently immunosuppress mice and induce susceptibility to disease has been used for other viral pathogens (30–33). The MAR1-5A3 clone mAb (hereafter referred to as MAR1 Ab) binds the mouse IFNAR1 protein and prevents dimerization with IFNAR2, thereby preventing recognition of Type I interferons by the Type I IFN receptor complex (INFAR-1/2 heterodimer) (34, 35). Here, we administered 500 µg of MAR1 Ab (or isotype control) 1 day before infection (−1 dpi) with OROV. Mice were monitored for survival until 28 dpi (Fig. 4A). Following MAR1 treatment, we found near-uniform lethality (95%) by 5 dpi at a dose of 10^6^ PFU OROV (Fig. 4B). At lower viral infection doses (10^5^, 10^4^, and 10^3^ PFU), mice succumbed to disease between 3 and 7 dpi in a dose-dependent manner with few survivors (Fig. 4B). We saw no lethality in isotype control-treated animals infected with 10^6^ PFU OROV, which was expected based on the existing literature and our previous findings (Fig. S1). MAR1-treated mice presented normally prior to rapid onset of disease, which was characterized by listlessness, hunching, piloerection, and abnormal breathing. Symptomatic mice quickly progressed to being unresponsive. Mice euthanized prior to succumbing to disease had high infectious OROV titers in gross liver homogenate (10^5^-10^8^ PFU/g; Fig. 4C), which correlated with high levels of viral RNA (Fig. 4D). In MAR1-treated mice, infectious virus and viral RNA were also detected in the spleen, kidney, lung, and brain at endpoint (i.e., time of euthanasia due to severe disease; Fig. 4E and F). Infectious titers in the liver were higher than in any other tested tissues.

**Fig 5:**
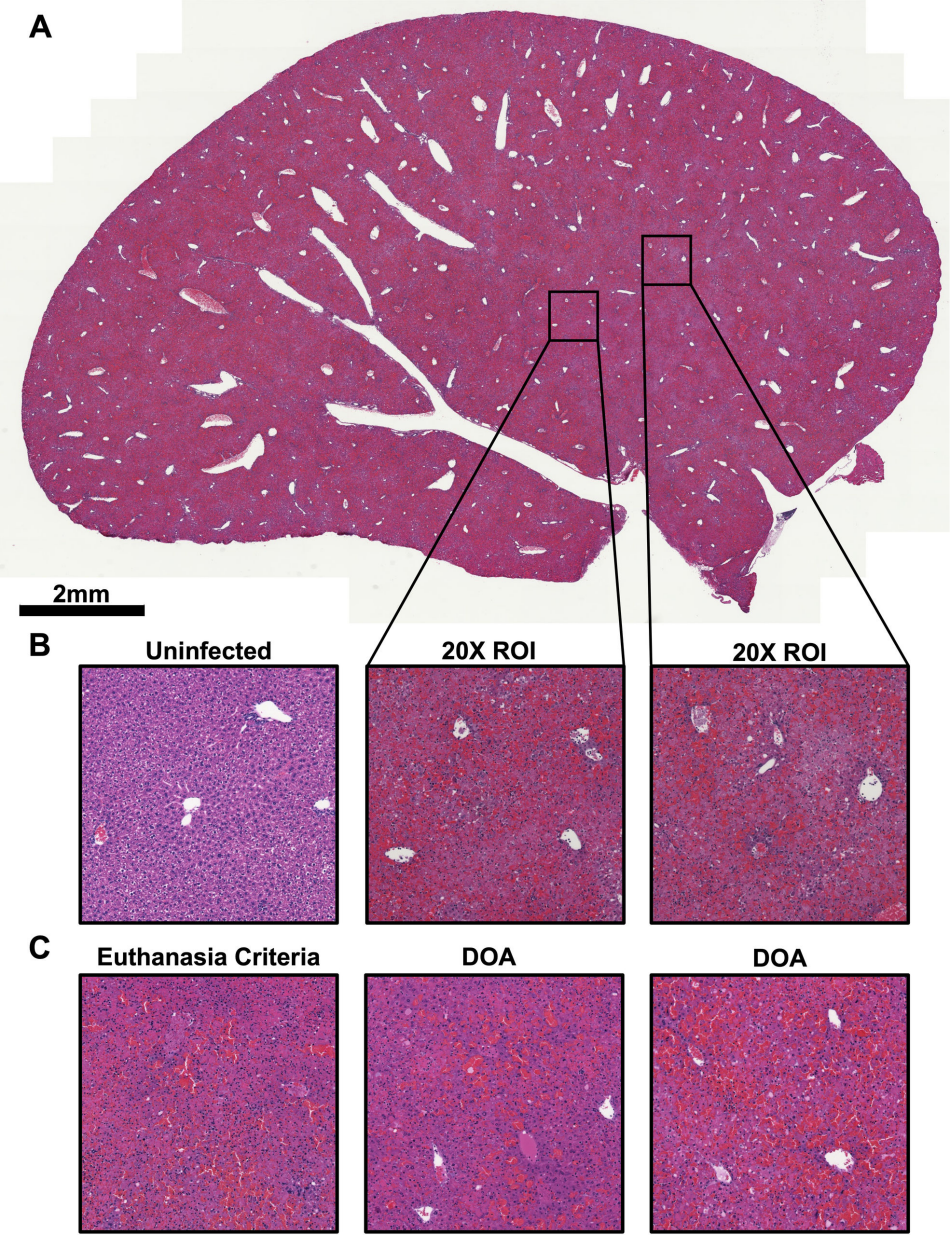
MAR1-treated mice have extensive hepatic damage at endpoint. (**A**) H&E staining of a representative FFPE liver left lateral lobe from a mouse treated with 500 µg MAR1 Ab 1 day prior to OROV infection with 10^3^ PFU and euthanized at a humane endpoint. Composite of 20× images. (**B**) Representative H&E 20× regions of interest (ROIs; 594 × 594 µm) from panel A and an uninfected control. (**C**) Representative H&E 20× ROIs (594 × 594 µm) from three different mice at endpoint after infection with 10^6^ PFU OROV and 500 µg MAR1 treatment. Labels indicate whether the mouse was euthanized at a humane endpoint or found dead on arrival (DOA).

Histopathologic examination of mouse livers at endpoint revealed fulminant hepatitis with massive hepatic necrosis across the whole lobule even at the lowest tested infection dose (10^3^ PFU; Fig. 5A). The cellular architectural damage, sinusoidal congestion, hemorrhagic necrosis, and large increase in infiltrating immune cells were consistent across all animals that succumbed to OROV disease (Fig. 5B). This pattern of pathology was observed in both mice euthanized at a humane endpoint and found dead on arrival (Fig. 5C). For mice that recovered from infection, there were no signs of histopathologic damage at 28 dpi (Fig. S8).

**Fig 6:**
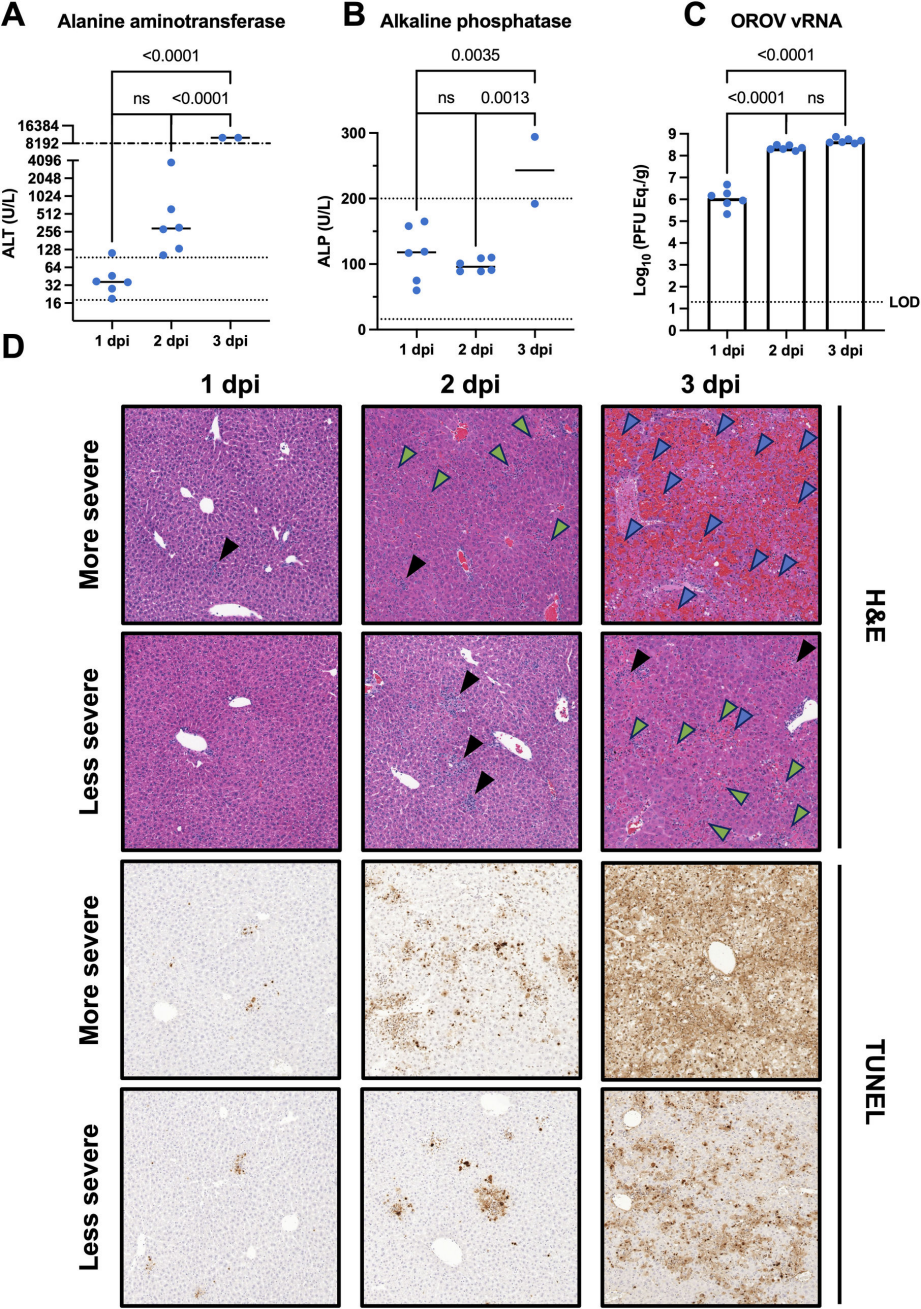
Liver injury in MAR1-treated mice is progressive and of rapid onset. Mice were treated with 500 µg MAR1 Ab 1 day prior to infection with 10^6^ PFU OROV via footpad injection. A subset of animals was euthanized at 1 (*n* = 6), 2 (*n* = 6), and 3 (*n* = 6) dpi. (**A**) Blood alanine aminotransferase (ALT) and (**B**) alkaline phosphatase (ALP) levels at indicated time points post-infection. (**C**) Viral RNA in liver homogenate from tissues collected at the indicated time point quantified by RT-qPCR. Dotted lines indicate either normal (95% confidence interval) range or limit of detection (LOD). Dashed line with dots represents upper limit of the assay. (**D**) Representative H&E and TUNEL 20× regions of interest (ROIs; 594 × 594 µm) from mice euthanized at 1, 2, and 3 dpi. Representative images have been partitioned into more severe or less severe based on pathologic interpretation. Black arrows mark necroinflammatory foci; green arrows mark bridging necrosis; blue arrows mark hemorrhage. Not all features are marked. Statistical significance was determined by ordinary one-way ANOVA and Tukey’s multiple comparisons test. *P*-value shown or ns (not significant) if *P* > 0.05.

Given the severe disease and liver damage seen at endpoint (3–6 dpi across all doses), we treated another group of mice with MAR1 Ab and conducted a timed euthanasia study at 1, 2, and 3 dpi after footpad infection with 10^6^ PFU OROV. At each time point, blood chemistry, viral titer, and histopathology were assessed. In line with our previous results (Fig. 4B), mice showed no clinical signs at 1–2 dpi, and 4/6 mice had succumbed to disease at 3 dpi. While there were no abnormal blood chemistry values at 1 dpi, by 2 dpi, hepatocellular damage was observed with elevated ALT and normal ALP (Fig. 6A and B and Fig. S9). At 3 dpi, four of six animals had succumbed to disease; therefore, blood chemistry analysis was performed only on the remaining two mice. We found that these mice had ALT values in excess of 10,000 ALT U/L, high ALP levels, and a myriad of abnormal blood chemistry readings suggesting extensive liver damage and likely multiorgan failure (Fig. 6A and B and Fig. S4). The blood chemistry values are in line with the massive hepatic necrosis seen at endpoint in our previous experiments (Fig. 4). Abundant viral RNA was detectable as early as 1 dpi in the liver of euthanized mice and exceeded 10^8^ PFU equivalents/g by 2 dpi (Fig. 6C). Infectious virus was detected in 3/6 livers at 1 dpi and increased to approximately 10^5^ PFU/g in all mice at 2 dpi (Fig. S10). At 3 dpi, while RNA titers increased further, infectious titers were lower (Fig. S10). Animals found dead on arrival or with extensive liver damage often have decreased infectious titers due to the high concentration of released liver enzymes and bile, which inactivate infectious particles. It is likely that the extensive damage observed at 3 dpi is responsible for the fall in infectious titers at this time point while RNA titers marginally increased.

Histopathologic examination of whole liver left lateral lobes revealed that some animals displayed significant histopathologic damage (labeled “more severe”) while others had less prominent damage (“less severe”; Fig. 6D and Fig. S11). This stratification aligns with the survival probabilities between 3 and 5 dpi where some animals are near death at 3 dpi, while others may live to 5 dpi (Fig. 4B). In more severe cases at 1 dpi, we found sparse necroinflammatory foci while less severe cases had no histopathologic signs of injury (Fig. 6D). At 2 dpi, more severe cases had signs of sinusoidal congestion, coagulative necrosis, and lipid accumulation, which is indicative of extreme stress (Fig. 6D). By contrast, the less severe cases at 2 dpi had necroinflammatory foci (Fig. 6D). At 3 dpi, less severe cases mimic the more severe cases at 2 dpi; however, more severe cases present as a fulminant hepatitis with massive necrosis across the entire lobule, as described above (Fig. 4 and Fig. 6D). This variability is likely the reason why approximately 45% of mice at this dose would survive past 3 dpi. As in the acute hepatitis model, ALT levels correlate with TUNEL staining (Fig. S12).

### Contemporary OROV outbreak isolate is less pathogenic in MAR1-treated mice

**Fig 7:**
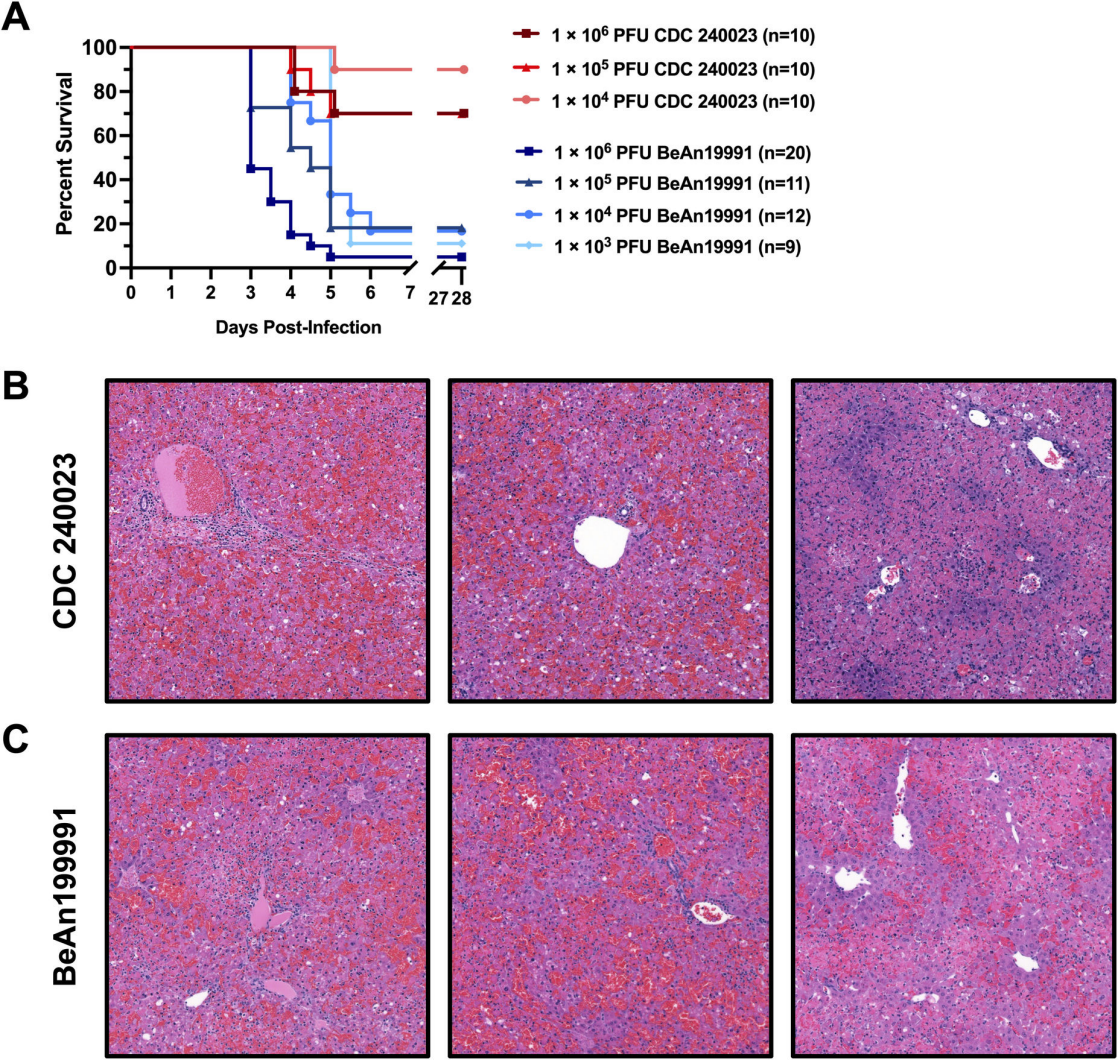
Contemporary OROV isolate is less pathogenic in laboratory mice than the prototypical strain. (**A**) Survival of mice treated with 500 µg MAR1 Ab 1 day prior to infection at the indicated OROV dose and strain via footpad injection. Representative H&E 20× regions of interest (ROIs) from mice that succumbed to (**B**) the contemporary OROV CDC 240023 isolate (*n* = 3) or (**C**) the recombinant prototypical OROV BeAn19991 (*n* = 52).

While the work with OROV BeAn19991 was ongoing, we received a more contemporary viral isolate (CDC 240023) from the ongoing outbreak; this isolate has been reported to be more pathogenic in Ifnar^−/−^ mice (36). OROV CDC 240023 is from the serum of a human patient who had recently traveled to Cuba (19). We evaluated the pathogenicity of OROV CDC 240023 in the lethal challenge model by treating mice with the MAR1 Ab one day before infection with 10^6^, 10^5^, or 10^4^ PFU. We found the contemporary CDC 240023 isolate caused markedly less lethality than the prototypical OROV strain (BeAn19991; Fig. 7A). We also found that the time to death was delayed by approximately one day with a compressed clinical window (4–5 dpi for CDC 240023 versus 3–6 for OROV BeAn19991; Fig. 7A). However, in the mice that succumbed to the contemporary OROV CDC 240023, we saw similar histopathology to the mice that succumbed to OROV BeAn19991, suggestive of fulminant hepatitis with massive hepatic necrosis (Fig. 7B and C). While these results suggest that OROV CDC 240023 may be less lethal under the experimental parameters used here, this does not negate the increased indices of severe outcomes seen in humans amid the ongoing outbreak. Rather, it suggests that current human isolates may be less adapted to infecting laboratory mice.

## DISCUSSION

Prior to the recent OROV outbreak in 2023, mouse models of OROV infection relied on either young animals (37, 38) or immunodeficient Type I IFN knockout (Ifnar^−/−^) mice to induce broad tissue susceptibility (39–41). Additionally, two recent reports noted that OROV appeared to be hepatotropic in mice (36, 42). Herein, we characterize two mouse models of OROV-induced hepatic disease. The first is a non-lethal model in immunocompetent mice which is characterized by transient, mild hepatocellular damage. The second is a lethal challenge model where mice are transiently immunocompromised using an anti-Ifnar1 mAb treatment. In both models, OROV infection is hepatotropic, which aligns with recent reports of lethal OROV hepatic disease in humans. In combination, these two models may prove useful to understand why most OROV infections are controlled and mild, while a minority progress to severe coagulative necrosis and liver failure. Both models will be useful in pre-clinical therapeutic and vaccine testing.

The non-lethal acute hepatitis model characterized here opens avenues to explore OROV-induced hepatocellular damage and repair from both immunological and histopathological lenses. The identity, abundance, and function of infiltrating immune cells in the necrotic foci were not explored in this study but may shed light on the mechanisms of OROV-induced tissue damage, clearance, and hepatic repair. Additionally, further understanding of how the liver responds to focal infection may shed light on liver injury and repair more broadly. To this end, characterizing the response at 2 and 4 dpi (in addition to 1, 3, and 5 dpi presented here) would be a worthwhile next step. The lethal challenge model presented here is advantageous for preclinical vaccination studies as it allows vaccination in an immunocompetent state prior to lethal challenge. Additionally, this model can be applied to existing transgenic mouse lines without needing to breed them in immunocompromised backgrounds.

**Fig 8:**
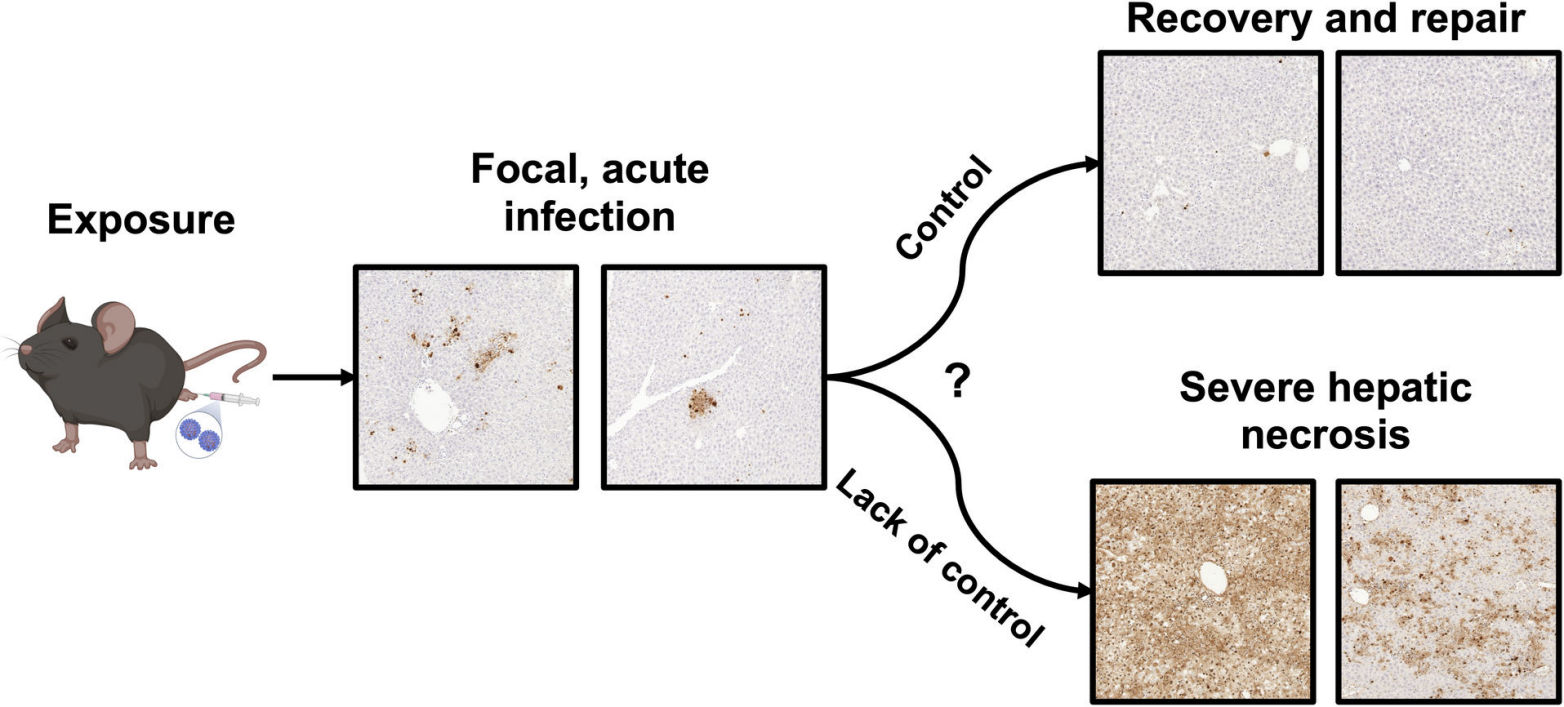
Model of liver pathogenesis caused by OROV. OROV infection in the footpad leads to focal infection of the liver. In immunocompetent mice, infection is controlled and repair follows (top). When the IFN response is suppressed, infection is uncontrolled and leads to massive necrosis and fulminant hepatitis (bottom). Some schematic ROIs duplicated from Fig. 3 and Fig. 6.

The similarity between ALT levels and histopathology of immunocompetent mice at 3 dpi and MAR1-treated mice at 2 dpi is striking. At these time points, we found mice have moderately elevated ALT levels ranging from the upper limits of the normal range to approximately 3,500 ALT U/L. Mice also have similar histopathology with necrotic foci sharing a similar distribution and characteristics at these time points. While mice at these time points appear to be experiencing similar disease manifestations, in the immunocompetent mice, the infection resolves and the liver repairs; whereas MAR1-treated mice progress to severe hepatic disease, necrosis, and death (Fig. 8).

While three lethal human cases of Oropouche fever provided detailed descriptions of liver histopathology, the third lethal human case also included representative microscopic images. The liver sections from this patient revealed severe hepatic damage, steatohepatitis, and necrotic foci which co-stained with OROV antigen, specifically in hepatocytes (26). This patient’s necrotic foci with infiltrating mononuclear cells look similar to the inflammatory foci we found in the sublethal C57BL/6 model at 3 dpi and the MAR1-treated mice at 2 dpi. Considering the cause of death in this case was determined to be renal failure with respiratory distress (despite a low level of OROV antigen in these tissues), it makes sense that the level of necrosis in the liver is more similar to the immunocompetent mice or MAR1-treated mice prior to progression in this study. Similarly, two recent human case reports from the current outbreak note liver enzyme elevation of a comparable magnitude and pattern to those observed in the mouse models shown here: that is, elevated ALT without elevation of ALP (27, 28).

The single dose of MAR1 antibody that we used was fivefold below the saturating level and yet it reliably induced OROV susceptibility (34). It is possible that lower doses of MAR1 may achieve the same outcome, or, conversely, higher doses with repeated maintenance doses may induce lethality at lower doses of OROV. Regardless, antagonizing the Type I interferon response with the MAR1 antibody leads to lethal disease, indicating that Type I interferons are a major OROV restriction factor, as previously reported (39, 43). Additionally, antagonism of the Type I interferon system enables OROV to infect non-hepatic tissues at detectable levels, which suggests that OROV has mechanisms to evade the initial Type I interferon response specifically in the liver. By comparing these two models, we may be able to determine what causes different outcomes (recovery or progression to severe hepatitis).

When comparing the prototype to a contemporary strain of OROV, the contemporary isolate OROV CDC 240023 was significantly less lethal in the MAR1 model than OROV BeAn19991, with an approximate one-day lag in pathogenesis for the mice that succumbed. Similar results were also observed in a recently published study (44). In contrast, it was also recently reported that OROV CDC 240023 had 100% lethality in Ifnar^−/−^ mice, with pathogenesis occurring 1 day earlier than BeAn19991 (36).

Subtle differences between the mouse models and challenge virus may account for these conflicting results. The BeAn19991 virus used here was generated by reverse genetics using the corrected sequence of the strain originally isolated in 1960 (45). While recovered using reverse genetics, BeAn19991 likely had a rich passage history prior to sequences being deposited. It is likely this included suckling mouse brain passages, which may have mouse-adapted BeAn19991 to some extent. In contrast, the CDC 240023 virus is the fourth passage of a recent clinical isolate from a human. It is not clonal and likely represents a mixed population. Further characterization of additional contemporary isolates, including with or without cloning and rescue, may be required to conclusively determine whether currently circulating strains are more virulent in mouse models. Additionally, lethality in a mouse model may not directly compare with virulence in humans.

In summary, the historically overlooked hepatotropic nature of OROV is now being appreciated due to the documented cases of lethal human disease with liver involvement, as well as careful study of laboratory animals with and without innate immune blockade. Understanding more about how OROV causes liver infection in both a lethal and sublethal context will be useful for evaluation of vaccines and therapeutic treatments such as small molecule inhibitors or monoclonal antibodies.

## MATERIALS AND METHODS

### Viruses

The prototypical strain of OROV (BeAn19991; GenBank accession numbers KP052850, KP052851, and KP052852) was generated by reverse genetics as previously published and generously provided by Dr. Natasha Tilston-Lunel (Indiana University) and W. Paul Duprex (University of Pittsburgh) (46). The stock used in this study represents the second passage in VeroE6 cells from the original rescue. The contemporary isolate of OROV (CDC 240023; GenBank accession numbers: PQ417950.1, PQ417949.1, PQ417948.1) was a generous gift from Brandy Russell (Arbovirus Reference Collection, Center for Disease Control and Prevention, Fort Collins, CO). The stock used in this study represents the fourth passage in VeroE6 cells (received at passage 2). OROV CDC 240023 was isolated by amplification in VeroE6 cells from the serum of a febrile human patient who contracted OROV in Cuba (19). All viruses were propagated in VeroE6 cells in D2 media (Dulbecco’s modified Eagle medium [DMEM] supplemented with 2% fetal bovine serum [FBS], 0.5 mg/mL Pen/Strep, 4.5 g/L glucose, 4 mM L-glutamine, and 110 mg/L sodium pyruvate). Viruses were concentrated by ultracentrifugation at 107,000 × *g* for 3 h using a Sorvall SureSpin 630 swinging bucket rotor in a Sorvall Discovery 90SE with a 25% sucrose cushion. Pelleted virus was resuspended overnight at 4°C, and then aggregates were removed by centrifugation at 3,000 × *g* for 10 min. Stocks were frozen in DMEM with a final concentration of 12% FBS, 0.5 mg/mL Pen/Strep, 4.5 g/L glucose, 4 mM L-glutamine, 110 mg/L sodium pyruvate, and 10 µM Tris Buffer (pH = 8.0). Viruses used to propagate concentrated stocks were sequence verified using the PCR-next-generation sequencing method previously described (47). OROV BeAn19991 showed no differences from the reference sequence. The OROV CDC 240023 had one mutation (L segment, nucleotide 222 T > C) and two polymorphic residues with approximately a 50% split in the population (L segment, nucleotide 1781 C > T; M segment nucleotide 2272 A > G). Stocks were titered by plaque assay.

### Animal studies

Experiments included both male and female mice to account for sex as a biologic variable. For antibody treatment, mice were intraperitoneally injected with 100 µL of sterile phosphate-buffered saline (PBS) containing 500 µg of either anti-Mouse IFNAR-1 [Clone MAR1-5A3], Purified *in vivo* GOLD Functional Grade (Leinco) or Mouse IgG1 GOLD, Clone hksp (Leinco). For infections, virus was diluted to the desired dose in D2 media. For footpad infections, 5- to 7-week-old mice were anesthetized with 2.5% vaporized isoflurane, then inoculated intradermally (ID) in the left footpad (20 µL per mouse using 29 G needles and insulin safety syringes). For intranasal infections, 3- to 4-week-old mice were inoculated using 50 µL split between both nostrils. For survival studies, mice were scored daily and then, if appropriate, euthanized at a humane endpoint as defined by the IACUC-approved euthanasia criteria or recorded as dead on arrival (DOA). For timed-euthanasia experiments, mice were sedated with isoflurane prior to euthanasia. Blood was harvested by terminal cardiac puncture, followed by harvesting of brain, liver, spleen, kidney, and lung tissue. Livers were dissected with the left lateral lobe being processed for histopathology, the right medial lobe being processed for immunofluorescent imaging, and the right lateral lobe and caudate process being homogenized for viral quantification. Tissues were homogenized in 0.5 mL D2 media using the Benchmark BeadBlaster 24 (6 m/s; 10 cycles; 30 s cycle/30 s pause).

### Virus quantification

Viral stocks and viral load in tissues were quantified by plaque assay on VeroE6 cells with a 3-day incubation. Viral load in tissue was also quantified by reverse transcription-quantitative PCR (RT-qPCR) quantified in PFU equivalents by generating a viral RNA standard curve targeting the genomic S segment from a stock of known titer. Homogenized tissue was diluted in D2 media for plaque assay or inactivated in TRIzol (Invitrogen) for RNA extraction and RT-qPCR. As described previously, we used a one-step RT-qPCR assay using primers/probe specific to the OROV S segment and detected genomic viral RNA (48).

### Blood chemistry

Blood chemistry analysis was performed using the VetScan VS2 Chemistry Analyzer (Zoetis) with either the Comprehensive Diagnostic Profile or Mammalian Liver Profile disks. Whole blood was harvested by terminal cardiac puncture and placed into lithium heparin tubes; tubes were rocked until blood could be analyzed. In cases where ALT values exceeded the dynamic range of the analyzer (>2,000 U/L), whole blood was diluted in 0.9% NaCl. In cases where insufficient blood was harvested or there were otherwise errors with the analyzer, those samples were excluded from presentation and analysis.

### Histopathology

Liver left lateral lobes were fixed in 10% neutral buffered formalin (NBF) then embedded in paraffin (FFPE) by the Cells, Tissues, and Models Core (CTMC) previously Clinical Biospecimen Repository and Processing Core at the Pittsburgh Liver Research Center (PLRC). The PLRC CTMC then performed hematoxylin and eosin (H&E) and TUNEL staining by standard methods. Microscopic histopathological interpretation was performed by Dr. Paul Monga (PLRC) blinded to the treatment groups. Brightfield images of stained slides were acquired using the Nikon PreciPoint Fritz Slide Scanner at the PLRC Advanced Cell and Tissue Imaging Core (ACTIC) within the Center for Biological Imaging (CBI; University of Pittsburgh) with a 20× Nikon CFI Plan Fluor objective. TUNEL staining was quantified using ImageJ by deconvoluting 3,3'-diaminobenzidine (DAB) pigmentation and thresholding TUNEL-positive area over the full tissue area. Thresholds were kept consistent across samples and experiments.

### Immunofluorescent staining

Liver right medial lobes were fixed in NBF, then cryo-embedded in O.C.T. compound (Fisher). Cryosections (5 µm) were permeabilized with 0.1% Triton X-100 made in PBS for 15 min. Tissues were washed with PBS and PBB (PBS supplemented with 0.5% bovine serum albumin) and then blocked with 5% normal goat serum for 1 h. A custom OROV N rabbit polyclonal antibody (GenScript) was diluted 1:200 in PBB and incubated with the tissue for 2 h. Subsequently, Phalloidin 488 (Invitrogen) and a goat anti-Rabbit antibody conjugated with AlexaFluor 647 (Invitrogen) were applied at 1:400 and 1:1,000 dilutions in PBB, respectively. Tissues were counterstained with Hoechst 33258 for 30 s and mounted using gelvatol provided by the PLRC ACTIC and CBI. Tissues were imaged using a Leica DMI8 inverted fluorescent microscope provided by the Center for Vaccine Research. All images were captured with the same settings and adjusted equally using ImageJ. A treated and untreated sample without primary antibody was used to adjust for background fluorescence.

### Statistics

For comparisons between blood chemistry values and viral titers, ordinary one-way analysis of variance (ANOVA) and Tukey’s multiple comparisons test were performed. Viral titers were log-transformed prior to analyses and graphing. For comparison of ALT levels and TUNEL area, a simple linear correlation and Pearson’s correlation coefficient were computed with two-tailed *P* calculation. *P*-values reported as exact or ns (not significant) if *P* > 0.05. Analyses were performed using GraphPad Prism (version 10.6.1).

## Data Availability

All data needed to evaluate the conclusions of this study are presented in the paper and its supplemental material. The data were stored in Microsoft Excel (version 16.106.2) and graphed using GraphPad Prism (version 10.6.1). Raw image files and full slide scans of histopathology are available upon request due to the large file size of each image.

## SUPPLEMENTAL MATERIAL

Supplemental figures, part I jvi.00611-26-s0001.pdf Fig. S1 to S6.

Supplemental figure, part II jvi.00611-26-s0002.pdf Fig. S7 to S12.

ASM does not own the copyrights to Supplemental Material that may be linked to, or accessed through, an article. The authors have granted ASM a non-exclusive, world-wide license to publish the Supplemental Material files. Please contact the corresponding author directly for reuse.
