# Supplementary material for: Oropouche virus causes acute hepatitis in mice controlled by type I interferons
Source: J Virol. 2026 Jun 30;100(7):e00611-26. doi: 10.1128/jvi.00611-26 (PMC13386938; doi:10.1128/jvi.00611-26)
Supplement: Supplemental figures, part I — Fig. S1 to S6. [file jvi.00611-26-s0001.pdf]

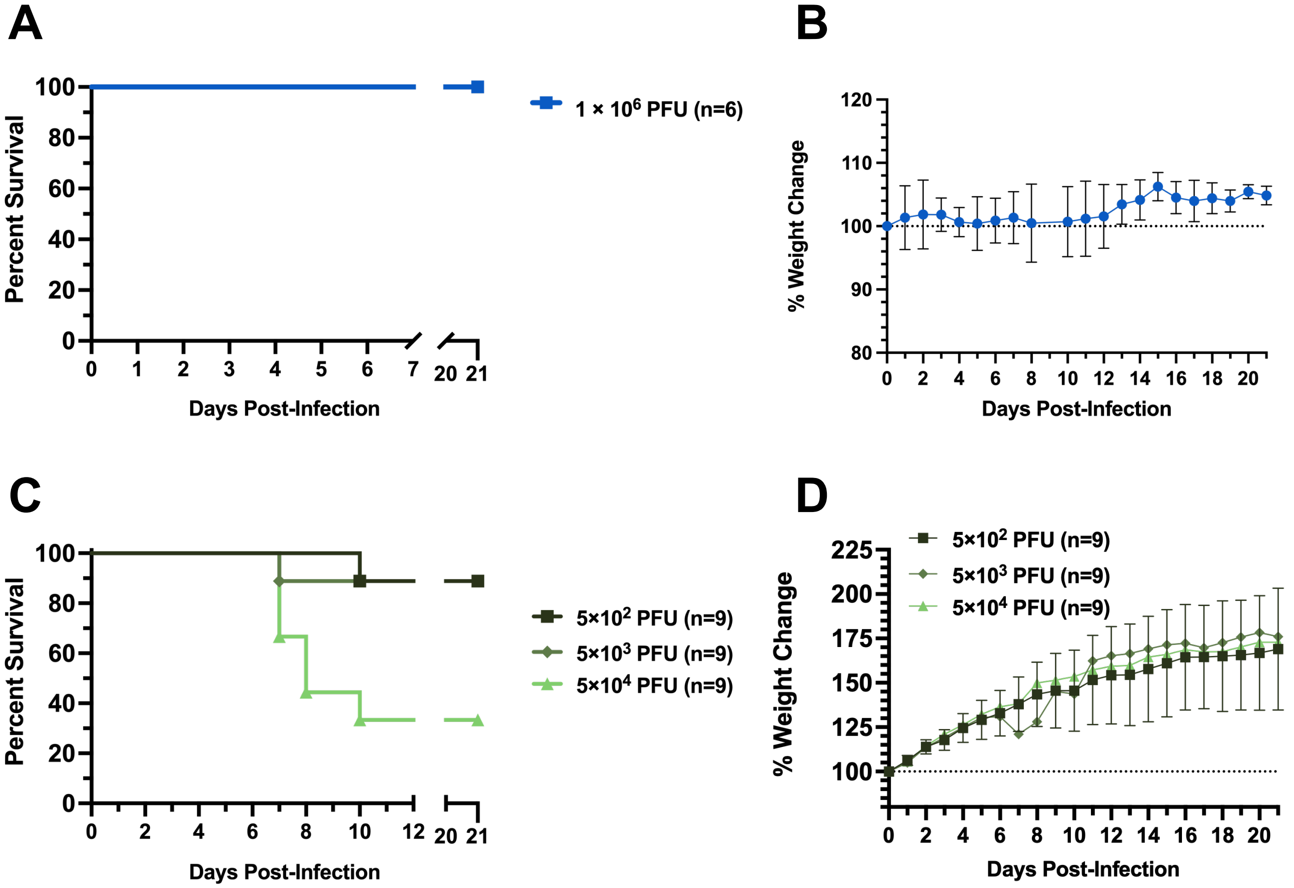

Supplementary Figure S1. Susceptibility of immunocompetent mice to OROV infection after footpad or intranasal inoculation. (A) Mice were infected with 10^6 PFU OROV via footpad injection (n=6) and monitored 21 days for signs of disease. (B) Mean mouse weight (normalized as percent change from baseline; n=6) with standard deviation error bars. (C) Mice were inoculated with the shown dose of OROV in 50 µL intranasally and monitored 21 days for signs of disease. (D) Mean mouse weight (normalized as percent change from baseline; n=9 per group) with standard deviation error bars.

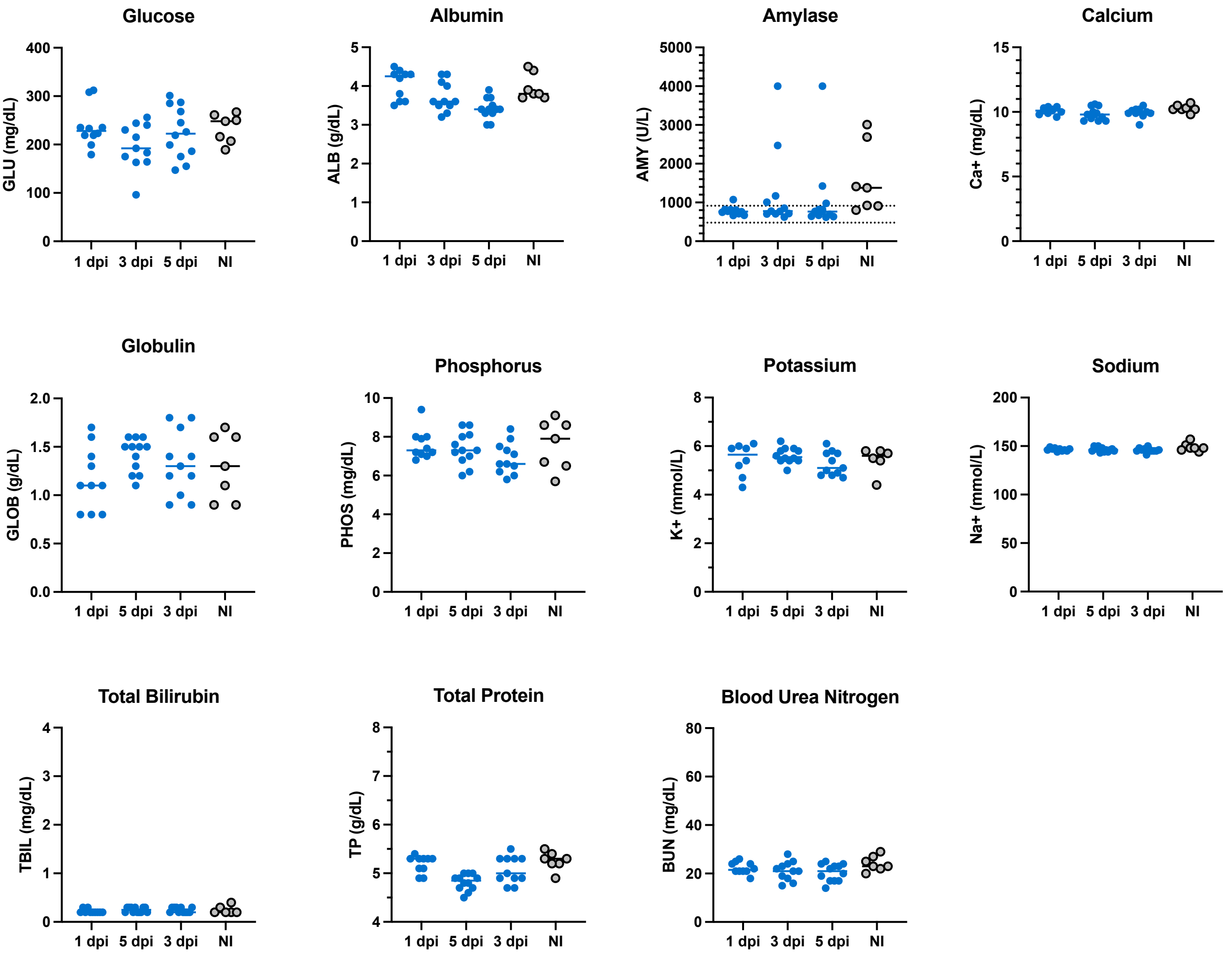

Supplementary Figure S2. Blood chemistry at 1, 3, and 5 dpi following OROV infection. Mice were infected with 10^6 PFU OROV via footpad injection and a subset were euthanized at 1 (n=11), 3 (n=15), and 5 (n=11) dpi. Uninfected controls were also included (n=7). Each data point represents one mouse across three experiments; bars represent median value. Dotted lines indicate normal (95 confidence interval) range. Each analyte and relevant unit shown on individual graphs. Uninfected controls labeled “NI” (not infected).

Figure S2

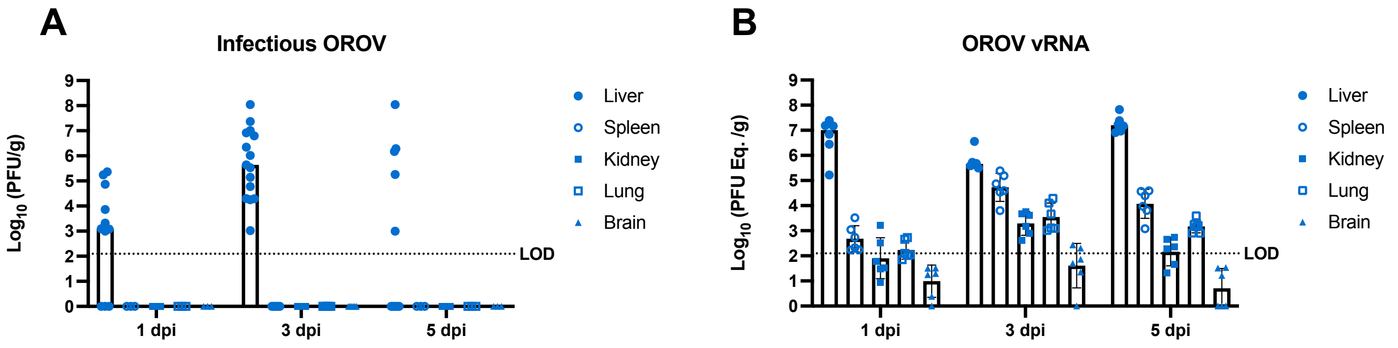

Supplementary Figure S3. Infectious titers and viral RNA at 1, 3, and 5 dpi following OROV infection. Mice were infected with 10^6 PFU OROV via footpad injection and a subset were euthanized at 1 (n=11), 3 (n=15), and 5 (n=11) dpi. (A) Infectious OROV titers by plaque assay or (B) viral RNA at indicated timepoints post infection and in indicated tissues. Each data point represents one mouse across three experiments; bars represent median value. For non-liver tissues, a subset (n=6 per timepoint) were quantified. Dotted lines indicate limit of detection (LOD) as defined by the tissue with the highest LOD.

Figure S3

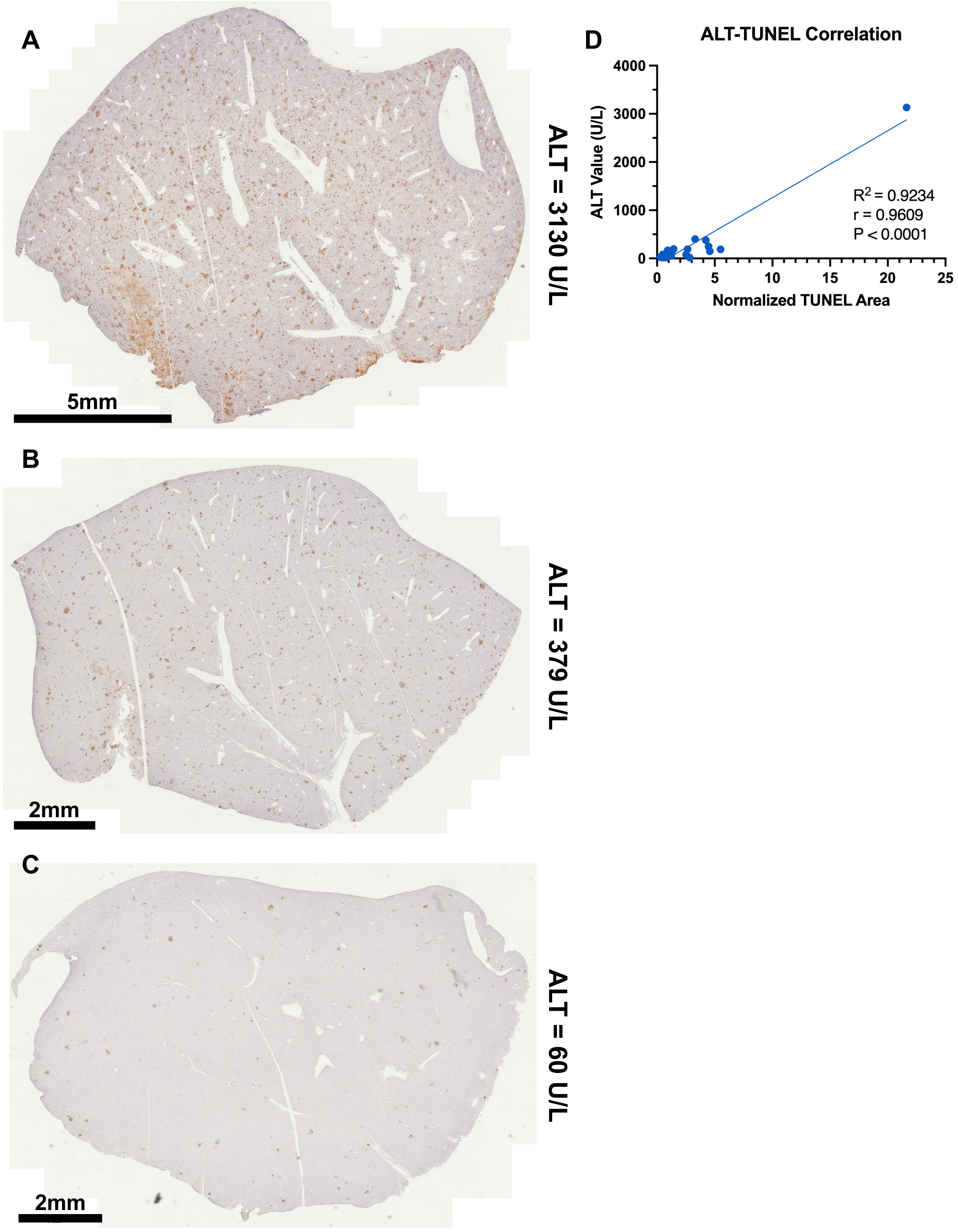
A
5mm
ALT = 3130 U/L
B
2mm
ALT = 379 U/L
C
2mm
ALT = 60 U/L
D
ALT-TUNEL Correlation
ALT Value (U/L)
4000
3000
2000
1000
0
0
5
10
15
20
25
Normalized TUNEL Area
R2 = 0.9234
r = 0.9609
P < 0.0001

Figure S4

Supplementary Figure S4. TUNEL area correlates with blood ALT values at 3 dpi following OROV infection. (A-C) TUNEL staining of representative FFPE liver left lateral lobes from mice euthanized at 3 days post infection (10^6 PFU OROV inoculation dose; total n=15, 3 independent experiments) and concordant ALT values. Composite images and a representative 20X ROI (594×594 µm) are shown. (D) Correlation between normalized TUNEL area (TUNEL area over full tissue area) and ALT value per animal. Each data point represents one mouse across three experiments (n=15). Simple linear regression and Pearson's correlation coefficient were computed.

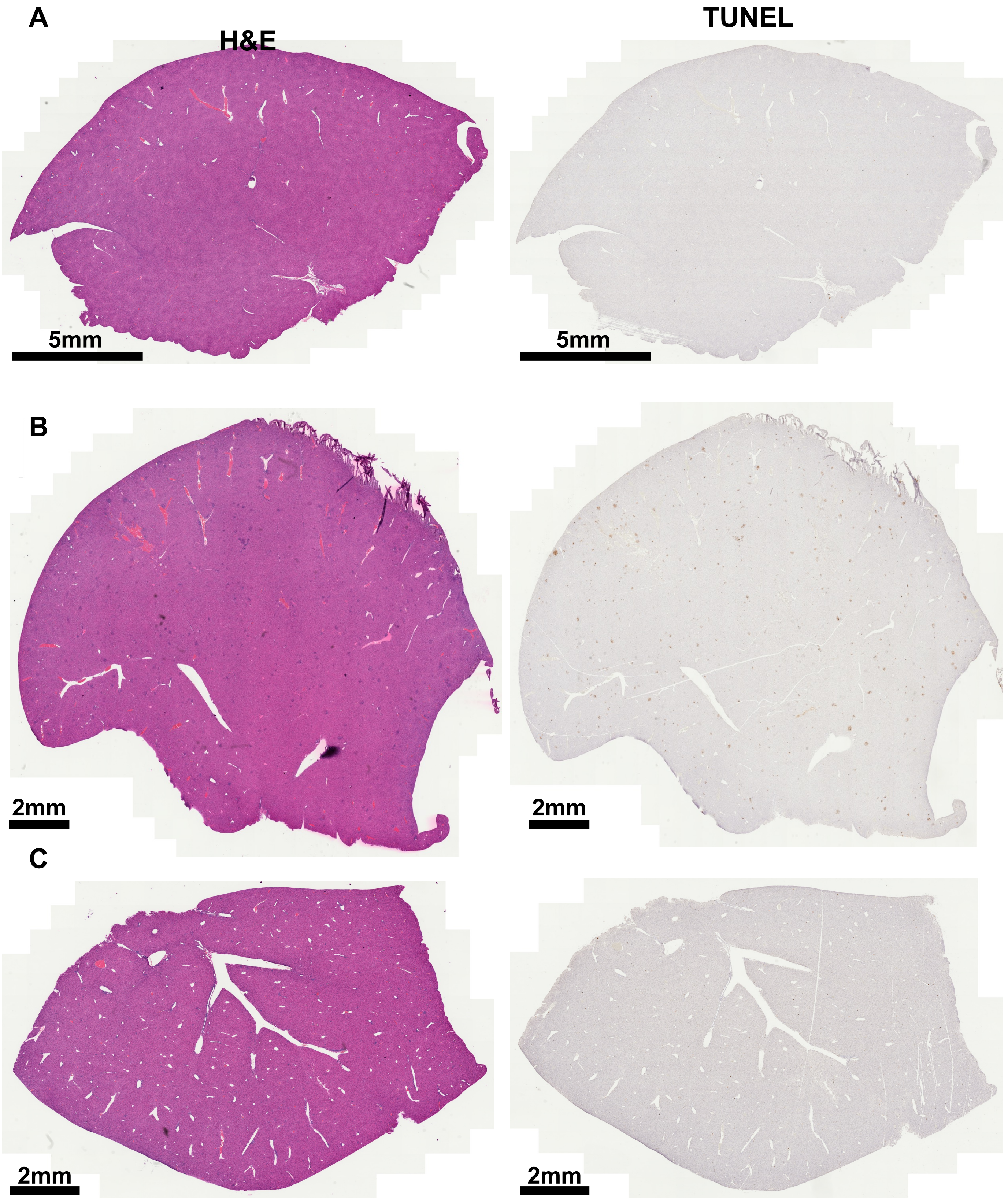

Supplementary Figure S5. Representative full lobe scans at 1, 3, and 5 dpi following OROV infection. H&E and TUNEL staining of a representative FFPE liver left lateral lobe from a mouse euthanized staining at 1 (A; n=11), 3 (B; n=15), and 5 (C; n=11) dpi.

Figure S5

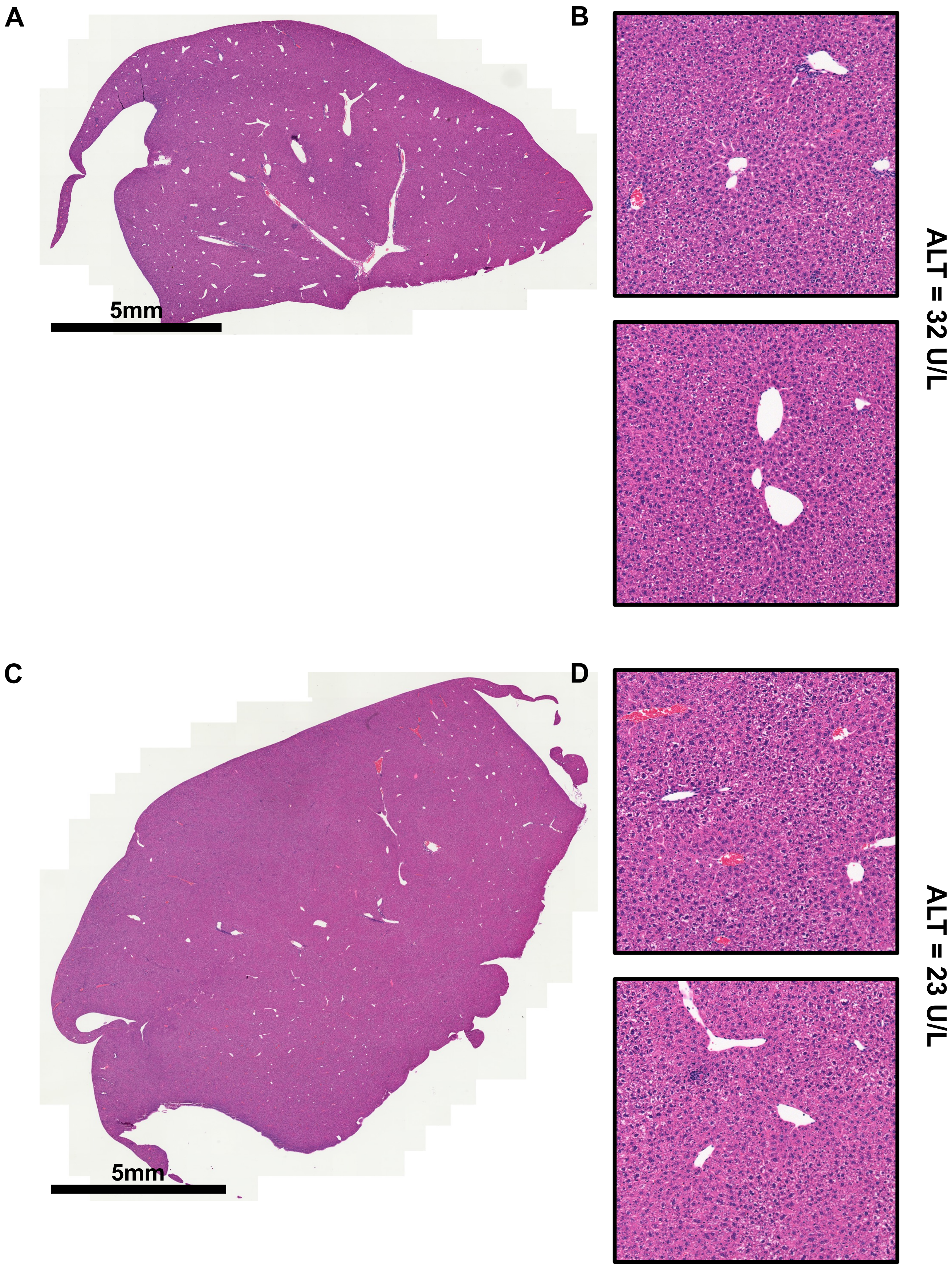

Supplementary Figure S6. Representative full lobe scans of uninfected control mice. H&E staining of representative FFPE liver left lateral lobes as full scans (A, C) or 20X ROIs (594×594 µm) from the same animal (B, D) with concordant ALT values.

Figure S6
