# Supplementary material for: Oropouche virus causes acute hepatitis in mice controlled by type I interferons
Source: J Virol. 2026 Jun 30;100(7):e00611-26. doi: 10.1128/jvi.00611-26 (PMC13386938; doi:10.1128/jvi.00611-26)
Supplement: Supplemental figure, part II — Fig. S7 to S12. [file jvi.00611-26-s0002.pdf]

A

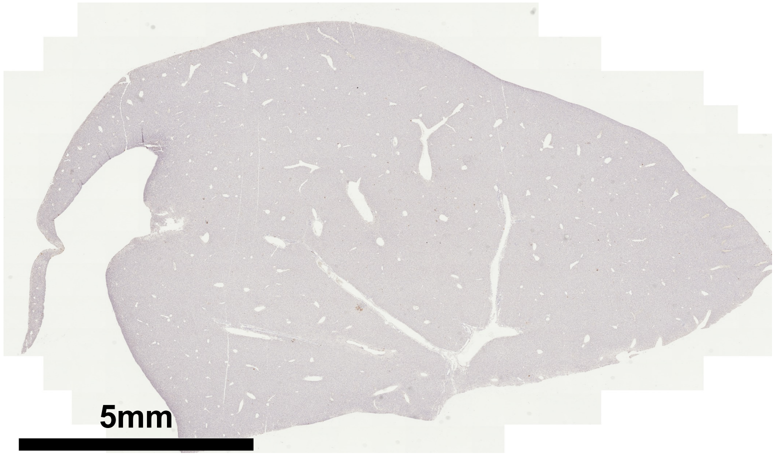

B

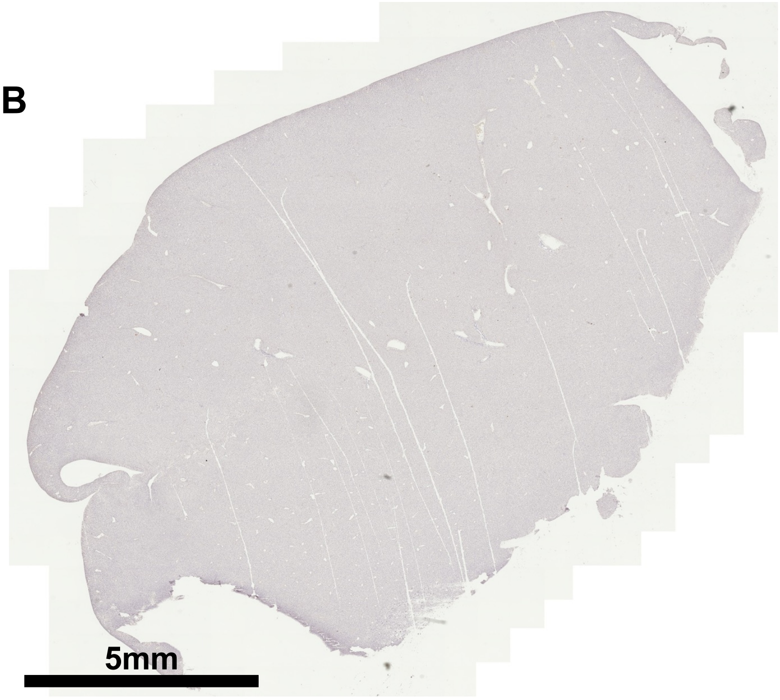

Supplementary Figure S7. Representative full lobe scans of uninfected control mice. TUNEL staining of representative FFPE liver left lateral lobes as full scans (A, B).

Figure S7

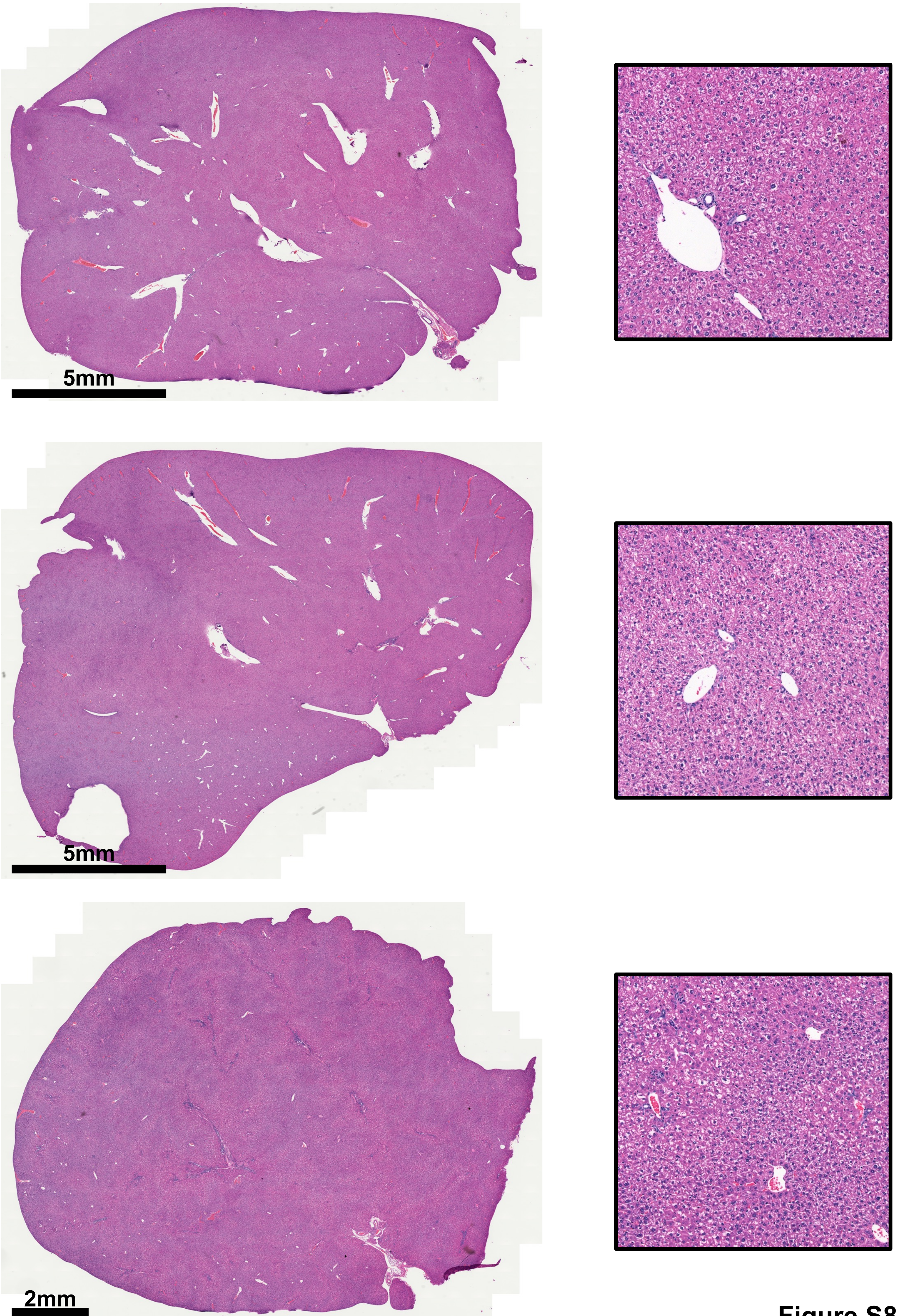

Figure S8

Supplementary Figure S8. Representative full lobe scans of MAR1 treated mice 28 dpi. Mice were treated with 500 µg MAR1 Ab 1 day prior to OROV infection via intradermal footpad injection. H&E of a representative FFPE liver left lateral lobes and 20X ROIs (594×594 µm) are shown from mice which survived to 28 dpi.

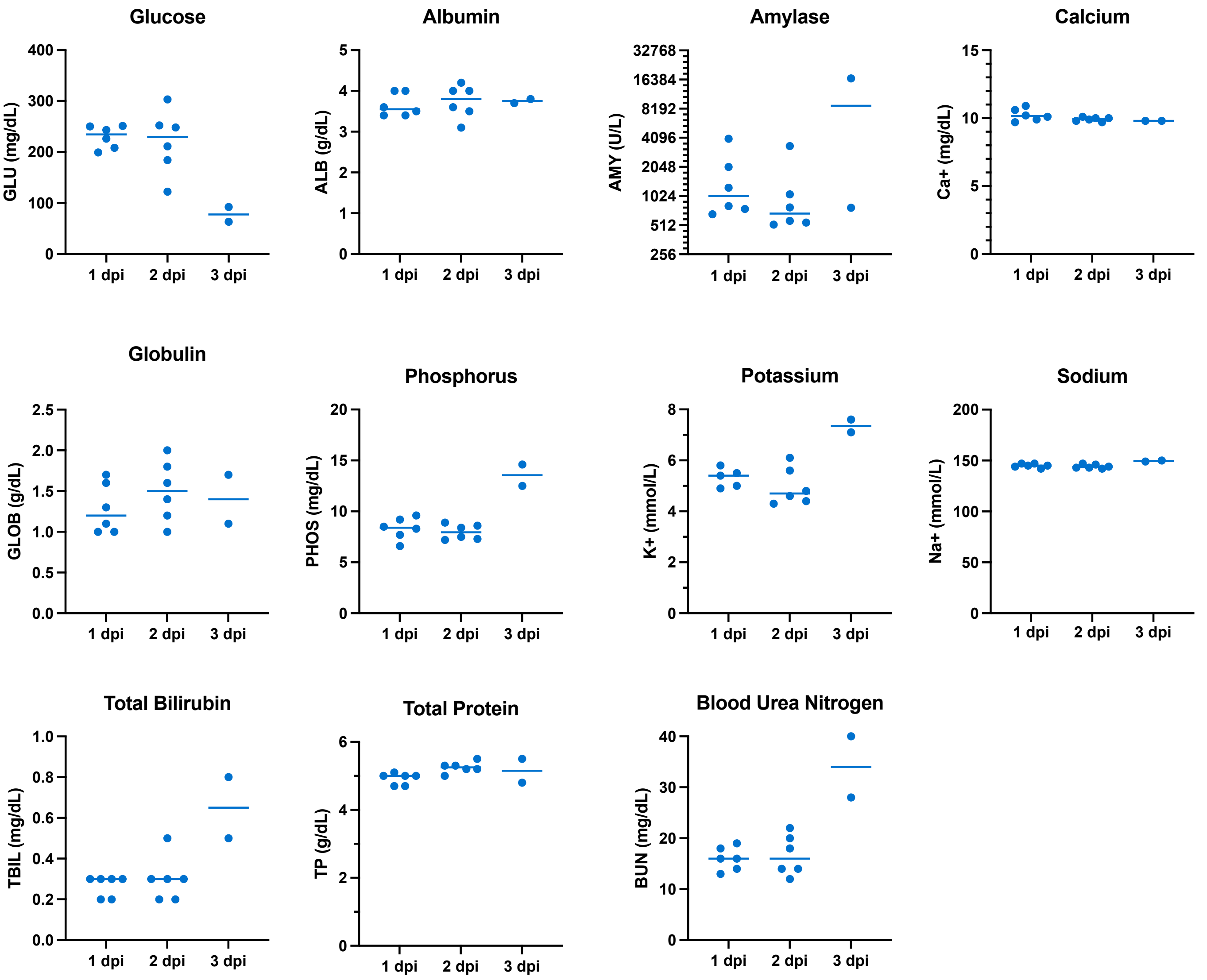

Supplementary Figure S9. Blood chemistry at 1, 2, and 3 dpi following OROV infection. Mice were treated with 500 µg MAR1 Ab 1 day prior to infection at 10^6 PFU OROV via intradermal footpad injection. A subset of animals was euthanized at 1 (n=6), 2 (n=6), and 3 (n=6) dpi. Each data point represents one mouse; bars represent median value. Dotted lines indicate normal (95 confidence interval) range. Each analyte and relevant unit shown on individual graphs.

Figure S9

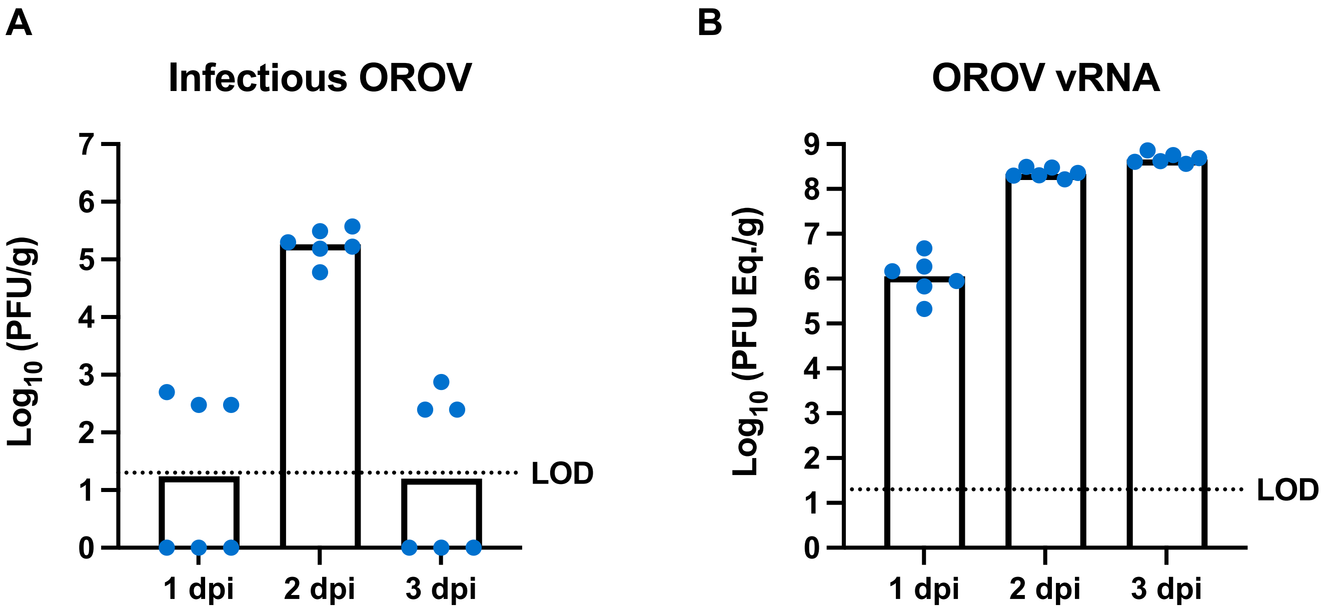

Supplementary Figure S10. Infectious titers and viral RNA at 1, 2, and 3 dpi following OROV infection with MAR1 treatment at -1 dpi. Mice were treated with 500 µg MAR1 Ab 1 day prior to infection at 10^6 PFU OROV via intradermal footpad injection. A subset of animals was euthanized at 1 (n=6), 2 (n=6), and 3 (n=6) dpi. (A) Infectious OROV titers by plaque assay or (B) viral RNA at indicated timepoints post infection and in indicated tissues. Each data point represents one mouse; bars represent median value. Dotted lines limit of detection (LOD).

Figure S10

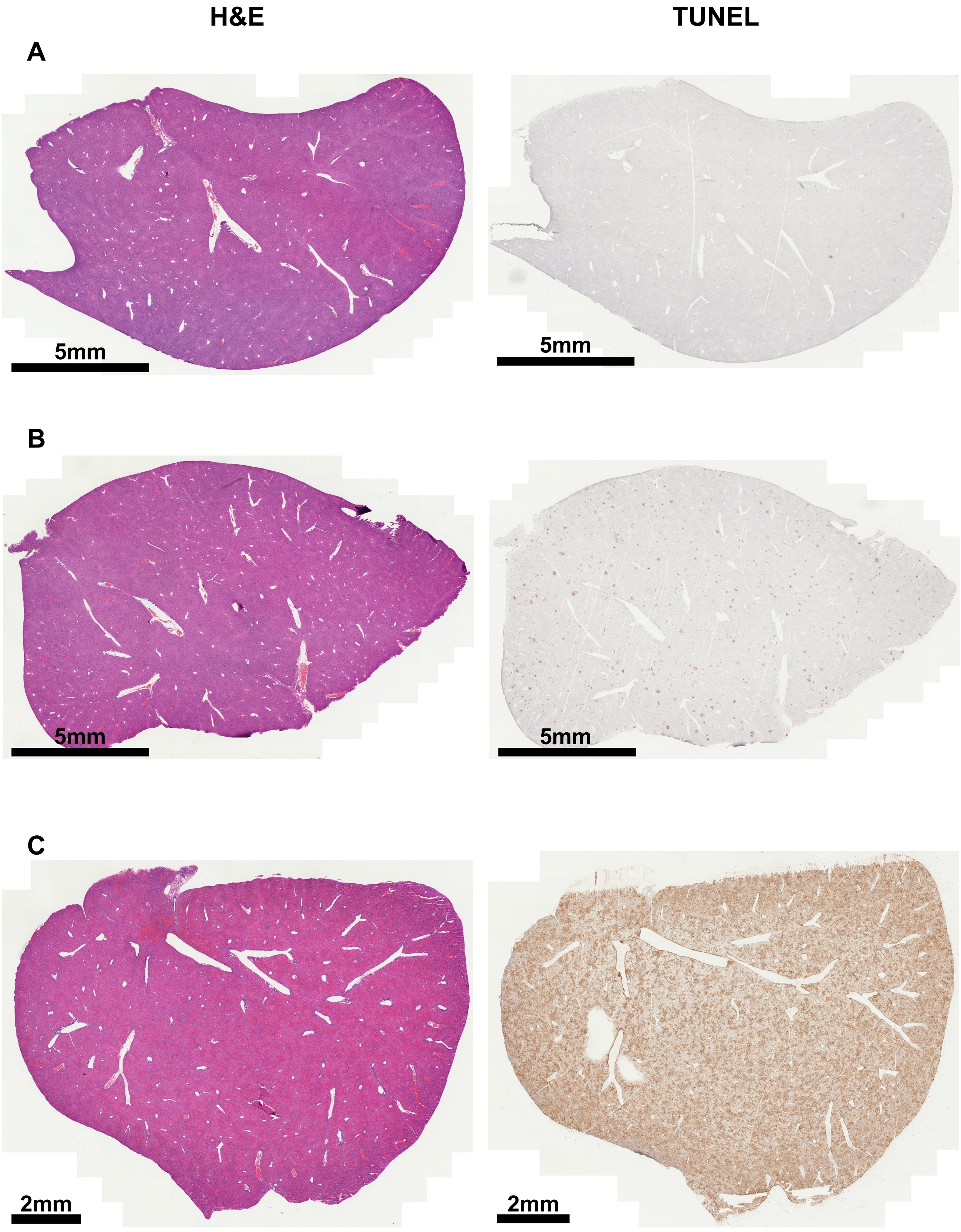

Figure S11

Supplementary Figure S11. Representative full lobe scans at 1, 2, and 3 dpi following OROV infection with MAR1 treatment at -1 dpi. Mice were treated with 500 µg MAR1 Ab 1 day prior to infection at 10^6 PFU OROV via intradermal footpad injection. A subset of animals was euthanized at 1 (A; n=6), 2 (B; n=6), and 3 (C; n=6) dpi. H&E (left) and TUNEL (right) staining of a representative FFPE liver left lateral lobe are shown.

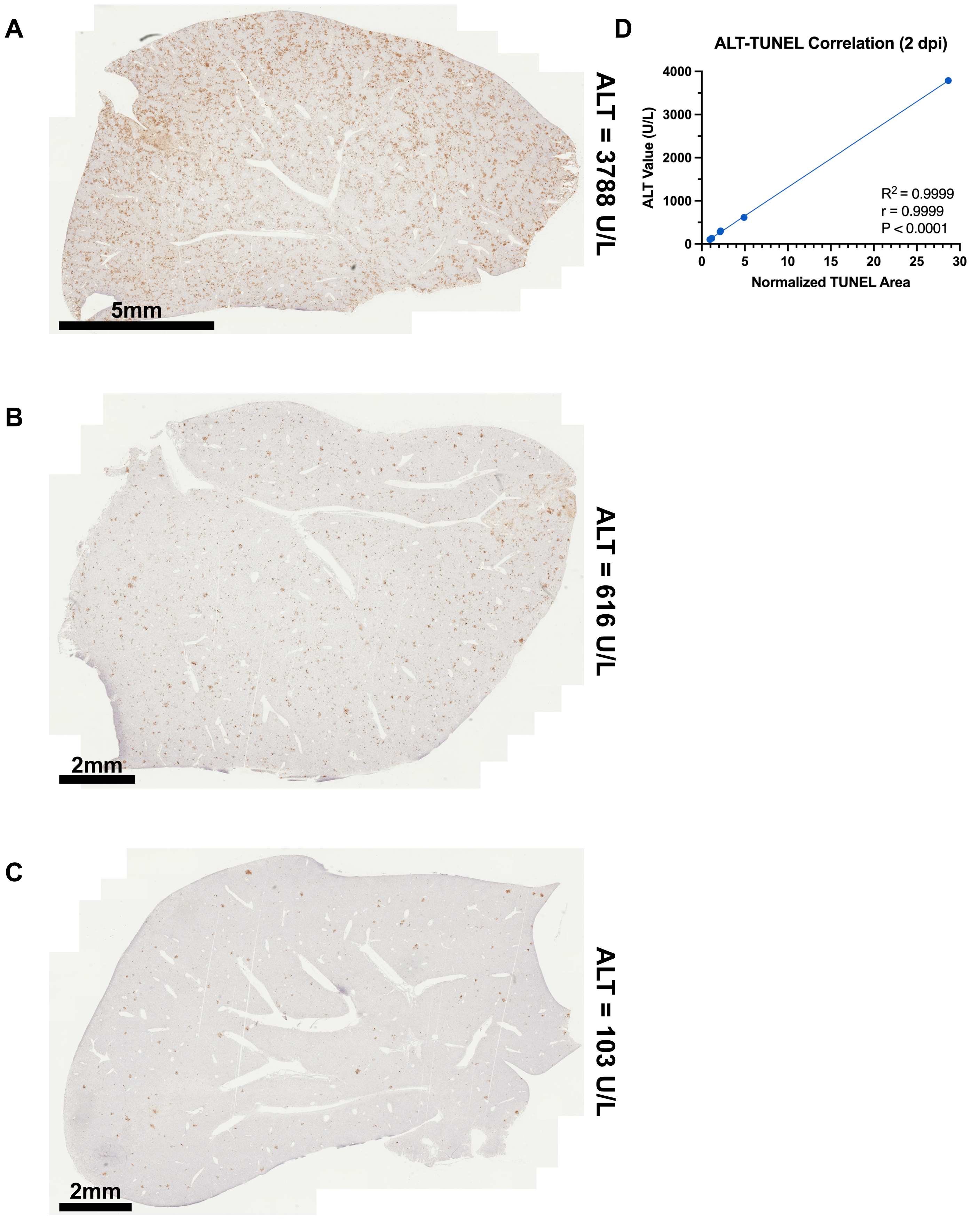

A
5mm
ALT = 3788 U/L
B
2mm
ALT = 616 U/L
C
2mm
ALT = 103 U/L
D
ALT-TUNEL Correlation (2 dpi)
ALT Value (U/L)
0
1000
2000
3000
4000
Normalized TUNEL Area
0
5
10
15
20
25
30
$R^2 = 0.9999$
r = 0.9999
P < 0.0001

Figure S12

Supplementary Figure S12. TUNEL area and blood ALT values correlate at 2 dpi following OROV infection with MAR1 treatment at -1 dpi. (A-C) TUNEL staining of a representative FFPE liver left lateral lobe from a mouse euthanized 2 days post infection (10^6 PFU OROV inoculation dose; n=6) and concordant ALT values. Composite images and a representative 20X ROI are shown. (D) Correlation between normalized TUNEL area (TUNEL area over full tissue area) and ALT value per animal. Each data point represents one mouse (n=6). Simple linear regression and Pearson's correlation coefficient were computed.
